# Allosteric modulation of AURKA kinase activity by a small-molecule inhibitor of its protein-protein interaction with TPX2

**DOI:** 10.1038/srep28528

**Published:** 2016-06-24

**Authors:** Matej Janeček, Maxim Rossmann, Pooja Sharma, Amy Emery, David J. Huggins, Simon R. Stockwell, Jamie E. Stokes, Yaw S. Tan, Estrella Guarino Almeida, Bryn Hardwick, Ana J. Narvaez, Marko Hyvönen, David R. Spring, Grahame J. McKenzie, Ashok R. Venkitaraman

**Affiliations:** 1 MRC Cancer Unit, University of Cambridge, Hills Road, Cambridge CB2 0XZ, United Kingdom; 2 Department of Chemistry, University of Cambridge, Lensfield Road, Cambridge CB2 1EW, United Kingdom; 3 Department of Biochemistry, University of Cambridge, 80 Tennis Court Road, Old Addenbrooke’s Site, Cambridge CB2 1GA.

## Abstract

The essential mitotic kinase Aurora A (AURKA) is controlled during cell cycle progression via two distinct mechanisms. Following activation loop autophosphorylation early in mitosis when it localizes to centrosomes, AURKA is allosterically activated on the mitotic spindle via binding to the microtubule-associated protein, TPX2. Here, we report the discovery of AurkinA, a novel chemical inhibitor of the AURKA-TPX2 interaction, which acts via an unexpected structural mechanism to inhibit AURKA activity and mitotic localization. In crystal structures, AurkinA binds to a hydrophobic pocket (the ‘Y pocket’) that normally accommodates a conserved Tyr-Ser-Tyr motif from TPX2, blocking the AURKA-TPX2 interaction. AurkinA binding to the Y- pocket induces structural changes in AURKA that inhibit catalytic activity *in vitro* and in cells, without affecting ATP binding to the active site, defining a novel mechanism of allosteric inhibition. Consistent with this mechanism, cells exposed to AurkinA mislocalise AURKA from mitotic spindle microtubules. Thus, our findings provide fresh insight into the catalytic mechanism of AURKA, and identify a key structural feature as the target for a new class of dual-mode AURKA inhibitors, with implications for the chemical biology and selective therapeutic targeting of structurally related kinases.

Aurora A kinase (AURKA) is a member of a family of Ser/Thr kinases whose orthologues control progression through mitotic cell division^1^,^2^. The Aurora family is evolutionarily conserved and three known human members of this family, Aurora A, Aurora B and Aurora C, bear sequence homology to those found in yeast and *Drosophila*^3^,^4^,^5^. The human Aurora kinases share a relatively conserved kinase catalytic domain at the carboxy-(C) terminus, but a small sequence variation within this domain contributes to the different spatio-temporal localization of the Aurora kinases during the cell cycle, leading to their distinct cellular functions^6^,^7^,^8^,^9^.

In particular, AURKA has been implicated in multiple events accompanying transit through the G2 to M phases of the cell cycle, and during mitotic cell division. These include centrosome multiplication^10^,^11^ and maturation^12^,^13^,^14^,^15^, bipolar spindle formation^16^,^17^,^18^, stability and function^19^,^20^,^21^. Indeed, while interphase cells contain a significant cytoplasmic pool of AURKA^22^,^23^, the kinase localises to distinct structures during cell cycle progression^24^,^25^,^26^. In late G2 phase, AURKA begins to accumulate at centrosomes, where it interacts with and is activated by numerous binding partners, such as Ajuba^27^,^28^, Arpc1b^29^,^30^, calmodulin^31^,^32^, CEP192^10^,^33^, NEDD9^34^ and nucleophosmin B23^35^. The centrosomal activation of AURKA is essential for pericentriolar material (PCM) accumulation and bipolar spindle formation. During prophase and metaphase, AURKA is recruited to the spindle microtubules, a less well-studied event essential for establishment of correct spindle length^10^. Upon anaphase onset, most AURKA is rapidly degraded by APC/C complex such that by the end of cytokinesis AURKA is largely undetectable^36^.

The localisation of AURKA to microtubules in the mitotic spindle is mediated through its interaction with a protein partner, TPX2^27^. The AURKA-TPX2 interaction involves the first 43 N-terminal amino acids of TPX2, which bind to the C-terminal catalytic lobe of AURKA. Crucially, mutation of three residues within this 43 amino acid stretch (Y8, Y10 and D11) abolishes the ability of TPX2 to bind AURKA, and leads to mitotic defects^10^. Besides localisation, the AURKA-TPX2 interaction modulates the catalytic activity of AURKA^37^,^38^. Recent structural and biological evidence separates the potential contributions towards AURKA activation made by activation loop auto-phosphorylation and TPX2-induced allosteric activation^24^. Upon binding, TPX2 induces an active conformation of AURKA as defined by internal hallmarks of active kinases: notably, the formation of a Lys-Glu salt bridge within the N-lobe, and hydrophobic spine alignment^39^. While the auto-phosphorylation of Thr288 of the activation loop results in a similar conformation, the TPX2 binding additionally protects the key phosphorylated Thr288 from solvent exposure and thus its accessibility to protein phosphatases^38^,^40^. The auto-phosphorylation appears to be essential in activating AURKA in the centrosomes at early stages in mitosis, and the allosteric activation by TPX2 of dephosphorylated AURKA may be critical during spindle formation.

Over-expression of AURKA occurs frequently in a range of human cancers^41^, inspiring attempts to therapeutically target its activity using ATP-competitive inhibitors now being tested in clinical trials^42^. These inhibitors have also proven invaluable for chemical biology studies of AURKA function^43^,^44^,^45^. However, ATP-competitive inhibitors are well known for exhibiting various levels of promiscuity^46^, prompting interest in the allosteric inhibition of AURKA by compounds that disrupt its interaction with the activator TPX2.

Here, we report the identification of AurkinA, a drug-like inhibitor of AURKA, which binds to a pocket occupied by Y8 and Y10 of TPX2 in the AURKA-TPX2 complex (the Y-pocket). AurkinA disrupts the AURKA-TPX2 interaction *in vitro*, and mislocalises the kinase from the mitotic spindle in cells. Unexpectedly, the binding of AurkinA to the Y-pocket leads to conformational alterations in AURKA distinct from those induced by TPX2 binding, restricting activation loop stabilization, and inhibiting catalytic activity via an allosteric mechanism. These alterations inhibit enzymatic activity *in vitro* and *in vivo*, but do not alter AURKA’s affinity for ATP, suggesting a novel mode of enzyme inhibition. Thus, our findings provide insight into the catalytic mechanism of AURKA, and provide a blueprint for the design of selective small-molecule inhibitors that target its Y-pocket, exemplifying a new allosteric approach for the dual-mode inhibition of AURKA localisation and activity.

## Results

### Identification and chemical optimisation of an Aurora-A-TPX2 inhibitor

**Figure 1:**
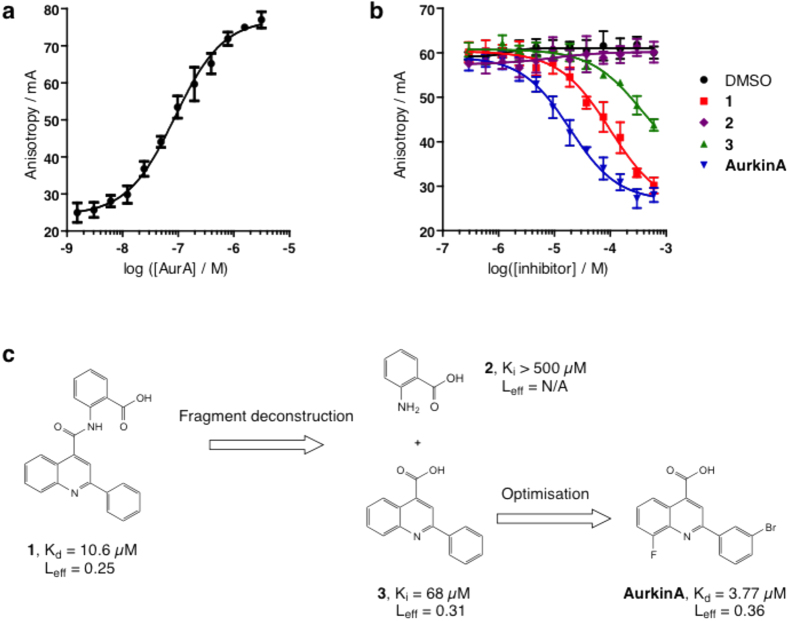
Identification of AurkinA, inhibitor of Aurora A-TPX2 complex. (**a**) The binding of AURKA_123–403_ to TAMRA-TPX2_1–43_ in fluorescence anisotropy assay. K_d_ = 83 nM (pK_d_ = 7.07 ± 0.07). (**b**) Displacement of TAMRA-TPX2_1–43_ by inhibitors in fluorescence anisotropy assay. (**c**) Synthetic strategy: deconstruction and optimisation of compound **1** identified from the HTS screen. K_i_ value was derived from FA competition assay using free-concentration corrected Cheng-Prusov equations^49^. K_d_ values were obtained using isothermal titration calorimetry. Ligand efficiency (L_eff_) was calculated using K_i_ or K_d_ values. Error bars represent standard deviation (n = 3).

We embarked on a high throughput screening (HTS) campaign using a library of 17000 rationally-selected compounds^47^ to identify compounds capable of disrupting the interaction between the AURKA catalytic domain and TPX2. We used a fluorescence anisotropy (FA) assay in which we measured the binding between a truncated recombinant His-tagged form of AURKA_123–403_ and a TAMRA-labelled TPX2 peptide fragment consisting of amino acids 1–43. The dose-dependent binding of the two components (Fig. 1a) allowed us to develop a statistically robust FA assay for screening (Supplementary Fig. 1).

The screen identified 15 potential inhibitors of the AURKA-TPX2 protein:protein interaction (PPI), resulting in hit rate of 0.09%. The HTS actives were resupplied and retested to check for dose-dependent inhibition of the PPI in FA assay. To remove the actives that bound to the ATP site of Aurora A and potentially allosterically inhibited the AURKA-TPX2 interaction, we performed a second round of the FA assay, with the addition of excess JNJ-7706621 (JNJ), a potent non-selective ATP-competitive Aurora A inhibitor^48^. JNJ did not interfere with the AURKA-TPX2 binding, and helped to stabilize AURKA protein (Supplementary Fig. 1). By comparing the activity of HTS hits in FA assay in presence or absence of the ATP site-blocking JNJ compound, we identified several compounds that were not significantly displaced by JNJ, suggesting they did not engage the ATP-site of AURKA (Supplementary Fig. 2). The most promising of these was **1** (Fig. 1c), which we chose to focus on.

The HTS hit **1** inhibited the AURKA:TPX2 interaction in a dose-dependent manner with an IC_50_ value of 41 μM (Fig. 1b). We further confirmed the direct binding of **1** to AURKA by isothermal titration calorimetry (ITC) (Supplementary Fig. 3). The ITC indicated that **1** bound to AURKA with a 1:1 stoichiometry, with binding affinity in the low micromolar range (K_d_ = 10.6 μM, pK_d_ = 4.98 ± 0.03). The direct binding affinity value was in agreement with the K_i_ value calculated from competitive FA assay (K_i_ = 8.9 μM) using the free-concentration corrected Cheng-Prusov equations^49^.

Fragment-based drug discovery has proved to be a useful tool in developing PPI inhibitors, permitting access to ligand-efficient hit elaboration campaigns^1^,^2^. With this in mind, we split **1** into constituent fragments, a process we refer to as fragment deconstruction (Fig. 1c). This yielded two simple fragments: **2** amino-benzoic acid, and **3**, 2-phenyl-4-carboxyquinoline, which is also known as cincophen, an approved veterinary drug used for the treatment of arthritis in animals. Unlike **2**, compound **3** caused a dose-dependent inhibition of TPX2 binding to AURKA in the FA assay (IC_50_ = 63 μM, Fig. 1b), suggesting that this was the critical part of the molecule that was responsible for the binding of H**1** to AURKA.

Accordingly, we synthesised new fragments exploring the structure-activity relationship (SAR) around the cinchophen moiety. Testing of the new set of elaborated fragments in the FA assay (Supplementary Table 1) revealed a number of SAR trends. Increasing hydrophobicity of the phenyl group increased potency in the FA assay, and introduction of a hydrophobic group, such as F, Cl, CF_3_ or Br at the *meta* position was particularly favourable. Interestingly, an isoquinoline variant of **3**, generated by moving the nitrogen, was poorly tolerated in the assay. This SAR insight allowed us to optimise the compound structure by iterative fragment synthesis and FA assay testing to yield AurkinA, a potent, binding-efficient, low-molecular weight inhibitor of the AURKA:TPX2 interaction (Fig. 1c). We determined the binding affinity of AurkinA to AURKA to be 3.77 μM (pK_d_ = 5.42 ± 0.03) by ITC (Supplementary Fig. 4), in line with its IC_50_ value in the FA assay. The stoichiometric association of AurkinA to AURKA was driven by enthalpy (∆H = −23.1 kcal/mol) and the binding was entropically disfavoured (-T∆S = 15.7 kcal/mol). The thermodynamic signature was consistent with an induction of a significant conformational change in protein structure upon the AurkinA binding^3^,^4^,^5^. The ligand efficiency of AurkinA was 0.36, which is considered to be a good metric for an early stage hit compound and on par with much smaller fragments, suggesting potential for further development^6^,^7^,^8^,^9^.

### AurkinA binds to a hydrophobic pocket in AURKA

**Figure 2:**
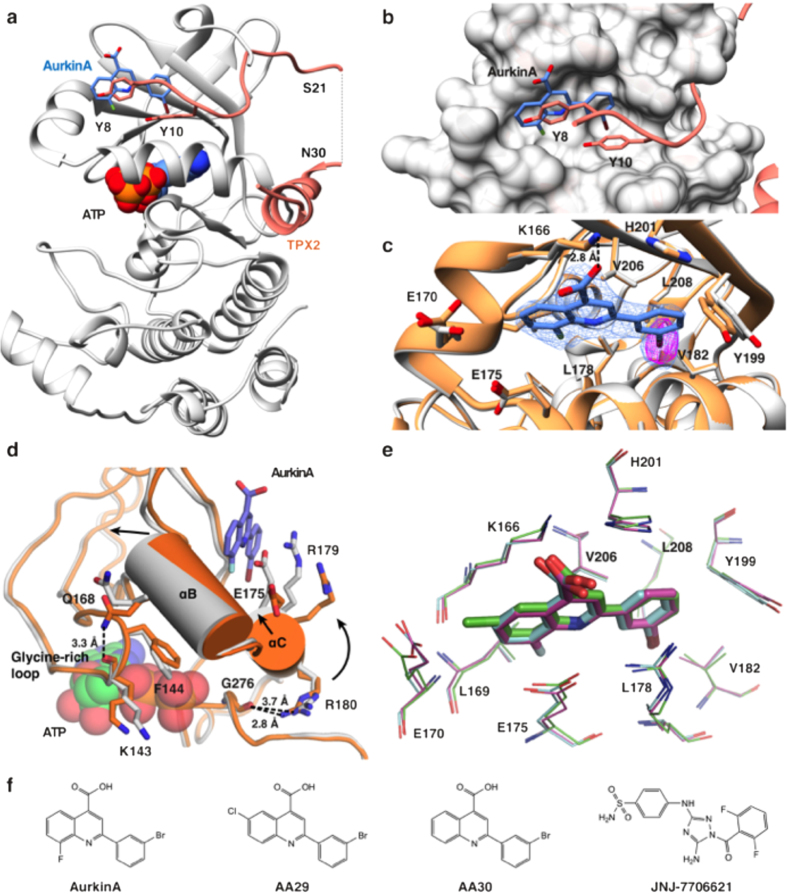
AurkinA triggers conformational changes in AURKA protein. (**a**) Crystal structure of AURKA_126–390_ liganded with Mg^2+^ -ATP and AurkinA (5DT4,gray), overlayed with TPX2_1–43_ (1OL5^38^, orange). AurkinA (blue) is bound to the pocket defined by αC and αB helices, a binding site of the YSY motif of TPX2. The hydrophobic Y-pocket sits above the ATP-site. (**b**) The detail of AurkinA (blue) and TPX2_8–11_ (orange) binding in the Y-pocket. (**c**) Binding pose of AurkinA in the Y-pocket. Carboxylic acid of AurkinA interacts with amine of K166, which is stabilised by H201. 2Fo-Fc map (blue) is countered at 1σ, anomalous map (pink) is contoured at 5σ. (**d**) The conformational change of Mg^2+^ -ATP liganded structure (5DT3, orange) upon AurkinA binding (5DT4, gray). The binding induces an anticlockwise rotation of αC helix by interacting with R179 and the hydrophobic floor. The shift of αB helix causes changes of hydrogen bonding pattern in glycine-rich loop, breaking of Q168-K143 hydrogen bond and movement of F144. (**e**) The overlay of AurkinA (magenta, 5DPV) and its analogues AA29 (green, 5DR9) and AA30 (cyan, 5DR6) bound to the Y-pocket. The related compounds bind with a consistent binding mode and the conformations of the amino acids lining the pocket are very similart between the liganded crystal structures. (**f**) The structures of the Y-pocket binders: AA29, AA30 and AurkinA; and JNJ-7706621, a potent ATP-competitive AURKA inhibitor.

To provide structural insight into how AurkinA binding might hinder formation of the AURKA-TPX2 complex, we determined the crystal structures of the AURKA catalytic domain in isolation or when liganded to AurkinA. Soaking of AurkinA into Mg^2+^-ATP-AURKA crystals yielded a liganded structure at 2.86 Å resolution (5DT4, Fig. 2, Supplementary Table 2). The electron density of AurkinA in the region of the pocket was well defined and its location and orientation was confirmed by an anomalous signal arising from the bromine atom at the *meta* position on the benzene ring (Fig. 2c, Supplementary Fig. 5). AurkinA was situated unambiguously in a hydrophobic pocket, lying in the groove formed by the αC and αB helices of the N-lobe (Fig. 2a). Comparison to the structure of AURKA in complex with TPX2 (Fig. 2b) demonstrated that this pocket accommodates both tyrosine residues within the YSY motif of TPX2, which has previously been shown to be crucial for the AURKA-TPX2 interaction^10^,^11^. We hereafter refer to this feature as the ‘Y-pocket’. Analysis of AurkinA’s binding pose in the Y-pocket suggests that it formed hydrophobic interactions between its quinoline and phenyl motifs and the hydrophobic floor of the pocket created by L178, V182, V206 and L208 (Fig. 2c). In addition, an ionic interaction was observed between the carboxylic acid of AurkinA and the basic side chain of K166. Of particular importance was the hydrophobic plug at the *meta* position of the benzene ring, which results in greater hydrophobic contact with the floor of the pocket. This observation was in line with the SAR data presented for the hydrophobic substituents at this position, demonstrating increased potency in the FA binding assay (Supplementary Table 1).

Interestingly, depending on the concentration of reducing agent in buffer, the AURKA kinase domain crystallised either in space group P6_1_22 or in P4_1_2_1_2 (Supplementary Table 2). In P4_1_2_1_2 crystals, grown under weakly reducing conditions, symmetry-equivalent C290 residues located on the activation loop formed an intermolecular disulphide bridge. The structure-stabilising bridge resulted in a more rigid structure, enabling us to obtain higher resolution crystals. We obtained AurkinA-liganded AURKA catalytic domain in 2.05 Å resolution (5DN3) confirming that AurkinA adopts a near-identical binding pose in the Y-pocket (Supplementary Fig. 8). However, the intermolecular disulphide bridge and crystal contact in proximity to the Y-pocket rendered a constrained structure and thus we used the lower-resolution P6_1_22 symmetry crystal (5DT4) to investigate conformational effects of ligand binding.

To further explore the structural characteristics of compounds that engage the Y-pocket of AURKA, we crystallised two further compounds (AA29, AA30) obtained as hits from our analogue screen (Fig. 2f, Supplementary Table 1). The overlay of the crystal structures of AURKA liganded to AurkinA, AA29 and AA30 respectively, demonstrated their conserved binding mode in the Y-pocket (Fig. 2e). We found that the ATP-site binding compound JNJ-7706621 stabilised the AURKA catalytic domain in solution (Supplementary Fig. 1c), and so we used it to assist the crystallisation process. We noted that whilst the structure for AA30, like that for AurkinA could be determined in complex with AURKA bound either to Mg^2+^-ATP or JNJ-7706621 in the active site, the AA29 compound could only be soaked into AURKA complexed with JNJ-7706621. Overall, the phased anomalous electron density for the bromine atom in the AA29, AA30 and AurkinA was stronger in the crystals with JNJ-7706621 (Supplementary Fig. 5).

### AurkinA induces conformational changes in AURKA

Analysis of the AURKA structure in hexagonal crystals showed that it remained relatively unchanged when the ATP binding pocket was occupied either with ATP or with JNJ-7706621. In contrast, the binding of AurkinA, and indeed any of our compounds increased the overall B-factors of the crystal structures (Supplementary Fig. 7). Importantly, in all structures where a small molecule is bound in the Y-pocket, the electron density for the AURKA activation loop remained largely undefined.

More detailed analysis of the liganded crystal structures revealed that binding of AurkinA and its analogues caused significant local alterations to AURKA structure, as well as changes at sites distant from the Y-pocket (Fig. 2d). Firstly, the αC helix (residues E175-S186) moved up by ~1 Å as measured between Cα atoms of R180 and R179 and was rotated anti-clockwise towards the N-terminal lobe. This resulted in formation of a π-cation interaction between the R179 side chain and AurkinA benzene ring, and a hydrophobic interaction between the L178 side chain and the quinoline moiety of AurkinA. The movement restricted the reach of R180, the residue that normally binds to phosphorylated T288 (pT288), as demonstrated by its inability to hydrogen bond with G276 in presence of the AurkinA. The R180-pT288 interaction is a key event in ordering of the activation loop, a first step towards the activation of the kinase^12^,^13^,^14^,^15^. Secondly, the binding of AurkinA in the Y-pocket caused an upward movement of helix αB (residues K166-A172, Fig. 2d). In turn, the αB helix shift introduced a movement in the ATP-binding glycine-rich (G-rich) loop, as signified by a shift of the residue F144 in presence of AurkinA. The hydrogen bond between Q168 on the αB-helix and the main chain carbonyl of K143 on the G-rich loop, observed consistently in the *apo* structures, was lost. In addition, a hydrogen bond between the backbones of F144 and F165 was significantly shortened from 3.2 Å to 2.8 Å upon AurkinA binding. These detailed observations are consistent with the overall changes in the dynamics of the activation loop. It has been proposed that the αC and αB helices, as well as the G-rich loop form a conserved unit that regulates AURKA kinase activity^16^,^17^,^18^. Collectively, these structural changes suggest that AurkinA binding in the Y-pocket might allosterically modulate AURKA kinase activity.

### AurkinA binding inhibits the kinase activity of AURKA in an ATP-independent manner

**Figure 3:**
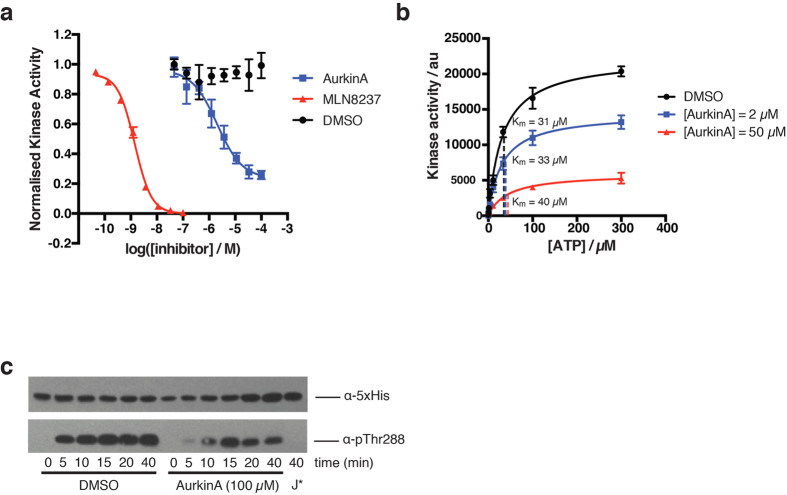
AurkinA causes allosteric inihibition of the kinase activity of AURKA. (**a**) AurkinA inhibits the kinase activity of pT287/pT288 AURKA_126–390_ in homogeneous time-resolved fluorescence assay (HTRF, KinEASY, Cisbio) in absence of TPX2. The volume of enzymatic reaction was 10 μL, [AURKA] = 5 ng/well, excess propriatory peptide substrate (1 μM) and ATP (100 μM). The reaction was incubated for 10 min. MLN8237 is an ATP-competitive inhibitor used as a positive control. AurkinA excess did not cause a complete ablation of AURKA kinase activity. (**b**) In presence of AurkinA, the maximum rate of AURKA kinase activity (V_max_) decreased, but K_m_ for ATP remained unchanged. Such a kinetic profile is consistent with a non-ATP competitive inhibition, suggesting that AurkinA inhibited the kinase activity of AURKA allosterically. (**c**) AurkinA slowed down the *in vitro* autophosphorylation of AURKA_GSMGS-126-390_ with uncleaved His-tag (30 μM), as detected by anti-pT288 antibody. [ATP] = 100 μM, J* = JNJ-7706621. Error bars represent standard deviation (n = 3).

To test this hypothesis, we used a commercially available *in vitro* kinase assay (KinEASE, Cisbio) to measure AURKA-dependent phosphorylation of a generic peptide substrate by homogeneous time-resolved fluorescence (HTRF) assay (Supplementary Fig. 9). Enzymatic phosphorylation occurred in the presence of the substrate peptide and ATP, and the phosphorylated substrate was subsequently detected in a proprietary antibody detection step. These studies were performed using a T287/T288 doubly-phosphorylated recombinant AURKA_126–390_ in the absence of TPX2. AurkinA treatment caused a dose-dependent inhibition of AURKA activity (Fig. 3a) and exhibited a low micromolar inhibitory constant (K_i_ = 3.7 μM), which was in line with its K_d_ for the recombinant AURKA. Interestingly, binding of AurkinA to the Y-pocket did not completely inhibit kinase activity, but instead, levelled out at ~75% inhibition. This is in direct contract to the ATP-competitive inhibitor, MLN8237. The presence of residual activity even at excess inhibitor concentrations (25x K_i_) raises the possibility that binding at the Y-pocket utilizes a distinct mode of AURKA kinase inhibition to ATP-competitive inhibitors, which achieve complete inhibition of the kinase.

We therefore carried out further kinetic experiments to determine the mechanism of ATPase activity inhibition by AurkinA and similar allosteric inhibitors of AURKA. Based on our structural data, we hypothesised that the allosteric, Y-pocket-binding inhibitors would not affect the K_m_ for ATP, as no significant changes in residues involved in ATP binding directly were observed upon AurkinA binding and ATP was always present in the active site. Instead, the structural data, which show changes in residues involved in ATP-ADP cycling and the activation loop ordering, suggested that the compound binding would alter the apparent turnover number of the enzyme (k_cat_) or its K_m_ for substrate. Indeed, the enzyme kinetics experiments revealed that addition of AurkinA at different concentrations did not change the apparent K_m_ for ATP while reducing V_max_ (Fig. 3b), a hallmark of non-ATP competitive binding^19^,^20^,^21^. This reduction in V_max_ is likely to incorporate the effect of compound binding on substrate affinity, since activation loop residues have been shown to interact with substrate in other kinases^22^,^23^. However, due to the assay’s intrinsic constraints on maximal usable substrate concentration, we were unable to fully separate the effects of compound on true catalytic turnover and K_m_ of substrate.

To further analyse the effect of AurkinA on AURKA kinase activity, we developed an *in vitro* auto-phosphorylation assay. It has been extensively reported that auto-phosphorylation of Thr288 in the activation loop stimulates AURKA kinase activity^24^,^25^,^26^. We therefore generated a non-phosphorylated form of AURKA (AURKA_GSMGS-126-390_, see methods) and used this to monitor the auto-phosphorylation over time by Western blotting with anti-pT288 AURKA antibody in presence or absence of AurkinA. As shown in Fig. 3c, AurkinA significantly delayed the auto-phosphorylation of AURKA, demonstrating the ability of Y-pocket binding to reduce AURKA’s kinase activity towards at least one of its physiological substrates. Importantly, these TPX2-free kinase activity assays show that AurkinA inhibited AURKA activity not by preventing TPX2-dependent activation as would be expected from a protein-protein interaction inhibitor, but by induction of unexpected structural changes which impact directly upon kinase activity.

In conclusion, the reduction of AURKA catalytic turnover is consistent with our structural data and provides a potential mechanistic link between Y-pocket binding and the inhibition of kinase activity.

### AurkinA mislocalises AURKA in mitotic cells and inhibits AURKA kinase activity

**Figure 4:**
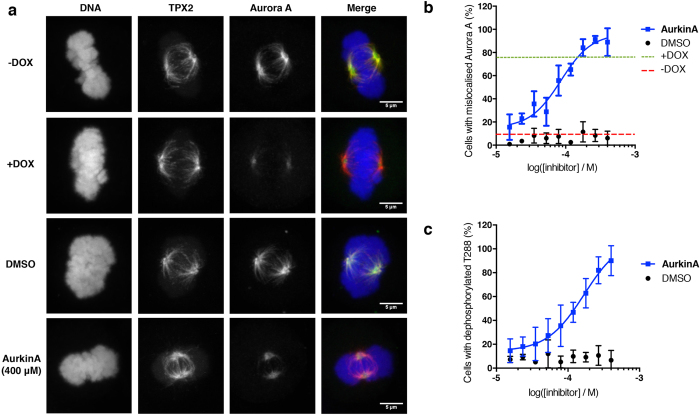
AurkinA mislocalises AURKA from the bipolar spindle in mitotic cells. (**a**) Representative immunofluorescence images of HeLa cells expressing mCherry-TPX2_1–43_ fusion protein under doxycycline (DOX) control. mCherry-TPX_21–43_ fusion overexpression as well as AurkinA mislocalise of AURKA from the spindle. (**b**) Quantitative analysis of AURKA mislocalisation from the spindle, as defined by % cells with below threshold AURKA intensity under TPX2 mask. AurkinA mislocalises AURKA from the spindle in a dose dependent manner with EC_50_ = 85 μM. (**c**) AurkinA deactivates AURKA, as measured by increase of % of cells with below threshold phosohorylated T288 (pT288). EC_50_ = 135 μM. The error bars represent standard deviation (n = 5).

Localisation of AURKA to the spindle during mitosis is dependent on binding to TPX2^27^,^28^. Overexpression of the AURKA-interacting TPX2 peptide has been shown to cause mislocalisation of AURKA from spindle microtubules in *Xenopus*^29^,^30^ and human cells^31^,^32^. The same effect was observed when key residues (Y8, Y10, and D11) in TPX2 were mutated^10^,^33^ or when an AURKA mutant (S155R) incapable of interacting with TPX2 was expressed^50^. In anticipation of observing this phenotype upon disruption of the AURKA-TPX2 interaction with a small molecule, we generated a HeLa cell line expressing inducible TPX2_1–43_ mCherry fusion (hereafter referred to as mCherry-TPX2_1–43_, Supplementary Methods). The cell line was validated by immunoprecipitation of mCherry-TPX2_1–43_ and subsequent immunoblotting for AURKA, which confirmed interaction between the TPX2 peptide and the endogenous AURKA protein (Supplementary Fig. 10a). Confocal fluorescence microscopy revealed that upon induction of mCherry-TPX2_1–43_, AURKA was mislocalised away from the spindle microtubules in mitotic cells (Fig. 4a). Based on this striking phenotype, we developed and validated an automated high-content microscopy assay with a Z’ of 0.51 (see Materials and Methods) to measure AURKA mislocalisation from spindle microtubules.

Exposure of cells to AurkinA phenocopies the effect of mCherry-TPX2_1–43_ expression, inducing mislocalisation of AURKA away from spindle microtubules in mitotic cells (Fig. 4a, bottom two panels). When this effect is quantified using the high-content assay described above, we observed a clear dose-dependent effect of AurkinA on AURKA localisation, with an EC_50_ of 85 μM (Fig. 4b). At higher concentrations of the compound, AURKA was almost undetectable on the spindle. Moreover, neither AurkinA nor the mCherry-TPX2_1–43_ affected the presence of AURKA at the centrosome (data not shown), which is in agreement with existing literature that TPX2 binding is not responsible for the centrosomal localisation of AURKA^10^.

Together, these observations provide strong evidence that AurkinA blocks the interaction between AURKA and TPX2 in cells. Since AurkinA can also inhibit AURKA kinase activity *in vitro*, we addressed the effect of the compound on AURKA kinase activity in cells by utilising a variation on the assay described above, measuring pT288 AURKA staining of the mitotic cells, instead of AURKA protein in general. Consistent with the *in vitro* assay results, AurkinA exposure causes a dose-dependent decrease in pT288-AURKA intensity (Fig. 4c). AurkinA therefore is able to both mislocalise AURKA, and inhibit its catalytic activity in cells.

## Discussion

Our findings demonstrate that AurkinA, a novel small-molecule inhibitor of the protein-protein interaction between the mitotic kinase AURKA, and the microtubule-associated protein, TPX2, inhibits AURKA localization and catalytic activity via an unexpected allosteric mechanism. AurkinA binds to AURKA primarily via interactions between its phenylquinoline core and the amino acid residues that line a hydrophobic pocket normally occupied by the Y8 and Y10 residues from TPX2 (the ‘Y-pocket’), with additional contributions from the *meta* phenyl hydrophobic plug and the polar interaction of the carboxylic acid moiety with the basic K166 and H201 residues that form part of the pocket. Residues involved in ATP binding are relatively unaffected by AurkinA engagement, suggesting an unusual mechanism for allosteric inhibition of AURKA. Consistent with this mechanism, we find that AurkinA does not alter the apparent K_m_ for ATP, although it reduces V_max_, features compatible with the non-ATP competitive mode of binding observed by crystallography. In cells, AurkinA caused a dose-dependent mislocalisation of AURKA from the mitotic spindle, to which it is normally recruited by TPX2. Moreover, AurkinA caused a dose-dependent decrease in T288 autophosphorylation, a cellular biomarker of AURKA activity. Collectively, these findings not only reveal a previously unrecognized, critical role for the Y-pocket in regulating the catalytic mechanism of AURKA, but also define it as a target for a new class of selective small-molecule inhibitors that act dually to inhibit AURKA localization and catalysis in cells.

The implications of our findings for AURKA catalysis are best understood in the context of two TPX2-dependent switches recently proposed to regulate catalytic activity^16^. Switch-1, the residue K143 situated within the G-rich loop, may play an essential role in the kinetic cycle of ATP binding, ATP hydrolysis and ADP release, which is controlled not only through an interaction between K143 and ADP, but also by the modulation of ADP binding by the hydrogen bond network between the αB helix and the glycine-rich loop. In addition, Switch-2, R180 of the αC helix, is known to capture an autophosphorylated residue - pT288 - of the activation loop in the active configuration of AURKA.

A previously unrecognized role of the Y-pocket in regulating AURKA activity is revealed by our crystallographic data comparing allosteric changes in the AURKA catalytic domain induced by the TPX2 peptide versus AurkinA. While TPX2 and AurkinA both bind to the same hydrophobic site, they affect the two AURKA kinase activity switches, K143 and R180, in the opposite ways. In a comprehensive structural study of Aurora-A:TPX2 interaction, Zorba *et al*.^24^ show the SYSYD moiety of TPX2_1–45_ opens the Y-pocket of inactive, dephosphorylated Aurora-A and forces the αC-helix to rotate clockwise (N to C). The rotation affects the position of R180 and makes it available for pT288 binding, which stabilises the activation loop, thereby increasing ATPase activity. Similarly, αB-helix is affected by TPX2 binding, resulting in the formation of a hydrogen bond between K143 of the ATP-binding, G-rich loop and E168. It has been suggested that this hydrogen bond facilitates the release of ADP from the ATP site by lifting the glycine-rich loop; indeed, we found it present in a number of structures of AURKA in the active conformation. The binding of the flat Y-pocket binders, exemplified by AurkinA, did not mimic the TPX2, but instead affected the AURKA switches in opposite ways. AurkinA binding caused the αC-helix to twist anticlockwise, which resulted in upwards movement of R180, restricting its range. Similarly, the hydrogen bonding interaction pattern between αB helix and G-rich loop was altered upon AurkinA binding. The F165-F144 backbone hydrogen bond was significantly shortened (3.2 Å to 2.8 Å) and the K143-Q168 hydrogen bond present in the ‘active’ apo structure was broken in the presence of the Y-pocket binders, potentially impairing the ADP removal from the ATP-site (see Supplementary Video 1). Taken together, these results demonstrate the crucial role of the αC-helix and the glycine-rich loop residues in the regulation of AURKA catalysis, and demonstrate the structural mechanism by which Y-pocket engagement by AurkinA allosterically modulates the kinase.

Importantly, our work also exemplifies a new approach for targeting AURKA and structurally related kinases for chemical biology or therapeutics development. AURKA shares with closely related AGC family kinases a high degree of structural similarity in the vicinity of the Y-pocket. Of note, PDK1 binds to its regulatory factor PIF using an analogous pocket (also called the ‘PIF pocket’), and several other therapeutically relevant AGC kinases (including the CMGC, TK or other families) also possess a Y- or PIF pocket. Thus, our studies not only add to the growing evidence that the catalytic activity of AGC family kinases can be modulated by small-molecule ligands that engage the Y- (or PIF) pocket, but also suggest that the approach we have taken may be more generally applicable.

## Methods

### Expression and purification of human Aurora-A kinase

All AURKA constructs (as described in Supplementary Methods) apart from AURKA_123–403_ were expressed in BL21 (DE3) cells containing pUBS550 plasmid cultured in 2xYT media supplemented with ampicillin (100 mg mL^−1^) and kanamycin (25 mg mL^−1^). Bacteria were grown at 37 °C until an OD_600_ of 0.5–0.6 when the temperature was lowered to 18 °C for 1 h before induction with IPTG (0.4 mM). Protein was expressed overnight at 18 °C and bacteria were harvested by centrifugation and pellets frozen in −80 °C. Thawn cells were resuspended to 5 mL/g final concentration in buffer A (50 mM Tris-HCl pH 8.0, 500 mM NaCl, 100 mM Mg(OAc)_2_, 1 mM ATP, 1 mM DTT, 40 mM imidazole) and lysed using Emulsiflex homogeniser. Lysate was cleared by centrifugation at 30000 × g at 4 °C for 30 minutes. Clarified lysate was loaded onto a HisTrap HP column (5 ml, GE Healthcare), washed with buffer A and eluted by buffer B (50 mM Tris-HCl pH 8.0, 500 mM NaCl, 100 mM Mg(OAc)_2_, 1 mM ATP, 1 mM MgCl_2_, 1 mM DTT, 600 mM imidazole). The N-terminal His-tag on the Aurora-A_GSMGS-126-391_ construct was cleaved off by incubation with His-tagged TEV protease at the molar ratio 1:30 and simultaneously dialysed against buffer A at 4 °C over night. Cleaved protein was loaded onto a 5 ml HisTrap HP column and eluted using a gradient of 40 to 120 mM imidazole. Eluted protein was gel filtered using HiLoad Superdex 75 16/60 column (GE Healthcare) equilibrated with buffer C (50 mM HEPES pH 7.4, 50 mM NaCl, 100 mM Mg(OAc)_2_, 1 mM ATP, 1 mM MgCl_2_, 1 mM DTT). Peak fractions were pooled, concentrated to 7 mg mL^−1^ and flash frozen in liquid nitrogen for storage at −80 °C. Protein concentration was determined by UV absorption at 280 nM using NanoDrop 1000 spectrophotometer (Thermo Scientific).

His-tagged AURKA_123–403_ construct was expressed by autoinduction in freshly transformed BL21 (DE3) cells with the pBAD plasmid. Starter cultures were initiated in LB broth supplemented with ampicillin (100 μg mL^−1^) and glucose (2%). After 4–6 h, large-scale ZYM-5052 cultures were inoculated from the starter cultures and incubated for 3 h at 37 °C followed by 16–18 h at 20 °C with 250–300 rpm shaking. The cells were harvested by centrifugation and pellets frozen in −80 °C. All following work was carried out at 4 °C. The pelleted cells were resuspended in binding buffer (50 mM Tris, pH 8.0; 40 mM imidazole, 500 mM NaCl, 5 mM MgCl_2_, 10% glycerol, 5 mM beta-mercaptoethanol, protease inhibitors) and lysed using Emulsiflex homogeniser. Lysate was cleared by centrifugation at 30000 × g for 45 min. The supernatant was loaded to equilibrated 2xHisTrapFF 5 mL colums at rate 0.3 ml/min column by Akta Purifier overnight, washed with wash buffer (50 mM Tris, pH 8.0, 40 mM imidazole, 1 M NaCl, 5 mM MgCl_2_, 10% glycerol, 5 mM beta-mercaptoethanol) and eluted with elution buffer (50 mM Tris-HCl, pH 8.0, 270 mM imidazole, 750 mM NaCl, 5 mM MgCl_2_, 10% glycerol, 5 mM beta-mercaptoethanol). The eluted protein was concentrated to 2.5 ml using a Vivaspin 20 (Sartorius) concentrator and buffer was exchanged to (20 mM Tris-HCl, pH 8.0, 500 mM NaCl, 5 mM MgCl_2_, 10% glycerol, 1 mM dithiothreitol) using PD10 column. The protein was further concentrated to 1 mL and loaded onto HiLoad 75 or 200 Superdex 16/60 column using Akta Purifier at 0.2 mL/min. Peak fractions were pooled, concentrated to 10 mg mL^−1^ and flash frozen in liquid nitrogen for storage at −80 °C.

### Isothermal Titration Calorimetry

Calorimetric titrations were performed on an iTC200 (MicroCal, Inc.) device. Protein was buffer exchanged into ITC buffer (Buffer C with 2% DMSO). Titration experiments were performed at 25 °C with an initial injection (0.4 μL) at a duration of 0.8 s, followed by 19 injections (2 μL) at a duration of 4 s. For the binding assays, compound **1** (600 μM) or AurkinA (300 μM) or was titrated into Aurora-A_G-126-390_ protein solution (30 μM). The spacing between injections was 180 sec and 300 sec for compound **1** and AurkinA, respectively. For the ITC experiments with compound **1** the correction for the heat of dilution was carried out by subtracting the average value of the last three data points of the titration. Correction for the enthalpy of dilution for the experiments with compound AurkinA was carried out by subtracting of blank injection heats obtained by the titration of the compound AurkinA solution into the ITC buffer. Binding isotherms were fit by non-linear regression using the single-site model provided by Origin software (MicroCal, Inc.). The stoichiometry of the interaction (N), equilibrium association constant (K_a_) and change of enthalpy (ΔH) were floated during the fitting.

### Auto-phosphorylation experiments

For autophosphorylation experiments Aurora-A kinase construct AurA_GSMGS-126-391_ was purified as described above but using buffers containing no ATP and His-tag was left on the N-terminus. To start the auto-phosphorylation reaction, ATP (100 μM) was added to the Aurora-A kinase samples in assay buffer (50 mM HEPES pH 7.4, 150 mM NaCl, 1 mM MgCl_2_, 1 mM DTT) at final protein concentration of 30 μM and incubated for up to 40 min. at room temperature in presence or absence of AurkinA (100 μM). 10 μL samples were taken 0, 5, 10, 15, 20 and 40 min. after adding ATP. The reaction was stopped by the addition of 10 μL 2× SDS-PAGE sample loading buffer. Control sample contained JNJ-7706621 (100 μM) that was added to the reaction prior ATP. 15 ng of each sample were run in 11% SDS-PAGE gels.

### Western blot analysis

Western blots were prepared by electro-blotting SDS-PAGE gels onto PVDF membranes (Millipore) and probing with anti-penta-his-tag antibody (1:2000 dilution, Qiagen, 34660) or anti-Aurora-A phosphoT288 antibodies (1:1000 dilution, Abcam, ab195748), followed by HRP conjugated TLCtmâ sheep anti-mouse antibodies (GE Healthcare) or HRP conjugated goat anti-rabbit antibodies (Abcam), respectively.

### Crystallisation and data collection

Aurora-A crystals were grown by hanging-drop vapour diffusion at 19 °C in crystallization buffer (100 mM HEPES pH 7.0, 200 mM MgSO_4_, 5–20% PEG3350). Crystals were cryoprotected in mother liquor supplemented with glycerol (20%) and DMSO (1–10%) and cryo-cooled in liquid nitrogen. For crystal-soaking experiments crystals were incubated over night at 19 °C in cryoprotecting solution supplemented with compound (1 mM).

Diffraction data were collected on beamlines i04–1, i02 and i03 at the Diamond Light Source (Harwell, UK) and Proxima 1 beamline at SOLEIL synchrotron (Saint Aubin, France) and were processed using Pipedream (Global Phasing Limited, A. Sharff, P. Keller). Data for crystals containing brominated compounds were collected at the wavelength near the bromine absorption edge (13500 eV) to maximise the anomalous signal. Manual rebuilding and model refinement was performed with COOT^51^, REFMAC^52^ and BUSTER^53^. Molecular topology was generated using PRODRG2 Server^54^ and the anomalous difference Fourier maps were generated using FFT^55^. Positive electron density peaks in the anomalous maps were used for unambiguous identification of the bromine compound location and binding stoichiometry. Molecular graphics images and “Morph movies” were produced using UCSF Chimera package^56^.

### Immunoblotting and co-immunoprecipitation

Stable HeLa FlpIn TREx cell line expressing a fusion mCherry-TPX2_1–43_ protein upon addition of doxycycline (0.5 mg ml^−1^) was generated (Supplementary Methods). This cell line was used for further experiments. For immunoblotting, proteins were extracted in lysis buffer (50 mM HEPES, pH 7.4; 100 mM NaCl, 0.5% NP-40, 10 mM EDTA, 20 mM β-glycerophosphate, 1 mM DTT, 1 mM sodium orthovanadate, 1 mM PMSF, protease inhibitors and phosphatase inhibitor (Roche)). 0.5 mg of lysate was used for co-immunoprecipitation. mCherry pull down was performed using RFP-trap (Chromotrap) following manufacturers’ instructions. Extracts were resolved by SDS-PAGE, transferred to PVDF membrane, blocked in TBS-Tween (50 mM Tris pH 7.6, 150 mM NaCl, 0.1% Tween-20) plus 5% non-fat dried milk, and probed with primary antibodies at 4 °C overnight. Membrane was incubated with secondary mouse HRP (GE Healthcare, NXA931) and rabbit HRP antibodies (Jackson Laboratories, 111-036-047) for 1 h at room temperature and the signal was developed using ECL or ECL prime reagent (GE Healthcare).

### Immunofluorescence (IF) and confocal microscopy

For confocal imaging, cells were grown on glass coverslips with appropriate treatments, fixed with ice-cold methanol for 10 min. Cells were permeabilised with 0.1% Triton-100 (Fischer), 0.1% Tween-20 (NBS Biologicals) in 1x PBS (PBS-Triton-Tween) for 10 min and blocked with 3% BSA (Fischer Scientific) in PBS-Triton-Tween for 30 min. The samples were incubated with antibodies: Aurora-A (1:1000) and TPX2 (Abcam 18D5–1, 1:500) diluted in blocking solution for 1 h at room temperature in humidified chamber. These was washed thrice in blocking solution, and incubated with Alexa Fluor- conjuagated secondary antibodies (Life Technologies) in dark for 30 min. Samples were washed with blocking solution, stained in Hoechst 33342, mounted in VectaShield^TM^ and stored in dark at 4 °C before microscopy. The images were captured using Leica SP5 confocal microscope using a 100 × 1.4 NA oil objective with z-stacks taken at 1 μm intervals. Pixel intensities were never saturated and laser exposure and detector settings were identical across an experiment to allow comparison between samples.

### IF staining, high-content microscopy and analysis

For identification of a single clone suitably expressing mCherry-TPX2 upon induction with doxycycline and low background in uninduced condition, the cells in 96-well plates were replica plated and grown in doxycycline (0.5 mg ml^−1^) containing medium for 24 h, fixed in formaldehyde (4%, v/v), stained for nuclei with Hoechst 33342 (4 mg ml^−1^) and imaged using high-content microscope - Cellomics ArrayScan^TM^ (ThermoFischer) and the intensity of mCherry expression was assessed using its Target Activation protocol.

For quantitative determination of Aurora-A mislocalisation from spindle microtubules, cells were plated at a density of 0.75 × 10^5^ cells per well of a clear flat bottom 96-well plate. On each plate a set of wells were treated with doxycycline as a positive control. After 24 h the compounds were diluted in 10 mM Bortezomib (Selleck Chemicals) and treated for 2 h. Hereafter all steps were carried out at room temperature. The cells were fixed by addition of freshly prepared PHEM-buffered formaldehyde (100 μL per well) for 10 minutes (100 mM PIPES, 20 mM HEPES, 5 mM EGTA, 2 mM MgCl_2_, 0.2% TritonX-100, 6.4% formaldehyde). These were permeablised with 100 μl of PBS-T-T (1X PBS/0.1% Triton- 100/0.1% Tween-20) for 10 min, blocked in 3% BSA-PBS-T-T for 30 min. Hereafter all the steps including dilution of antibodies was done in the blocking solution. A cocktail of primary antibodies (50 μL, mouse anti-TPX2, Abcam 32795, 18D5-1, 1:500; rabbit anti-Aurora A, Millipore EP1008Y, 1:1000) for 1.5 h and washed thrice with the blocking solution. A cocktail of secondary antibodies (50 μL, goat Alexa Fluor 633 anti-mouse IgG, Life Technologies, A21052, 1:500; goat Alexa Fluor 488 anti-rabbit IgG, Invitrogen, A11034, 1:500) and incubated for 30 min in dark. The cells were washed and the DNA was stained by Hoechst 33342 (4 μg ml^−1^) in 1X PBS. Removed excess stain by washing twice with 1X PBS and added 100 μL 1X PBS. Plates were stored at 4 °C in dark before scanning on Cellomics ArrayScan (ThermoFischer).

The plates were scanned using Molecular Translocation protocol with 40X objective with 3 flurophore recognising channels. Channel 1 was used to identify Hoeschst-stained nuclei as primary objects, channel 2 was used to identify TPX2-stained spindles in mitotic cells amongst valid channel 1 objects, and channel 3 was used to identify Aurora-A staining in valid channel 2 objects. The software generated an overlay of TPX2-stained spindles (channel 2), and the average intensity of the Aurora-A (channel 3) within that mask was scored. After the plates were scanned, the data was exported into Excel and the highest Aurora-A intensity in the darkest 10% of untreated samples was used to set a threshold below which Aurora-A was classified as delocalised. Once the threshold was applied, the percentage of cells with Aurora-A intensity in the TPX2 mask below the threshold was reported as percentage of cells with mislocalised AURKA.

### High-content AURKA T288-dephosphorylation assay

A modified version of the Aurora A mislocalisation assay using the same cells, doxycycline-treated controls, drugging plan, Bortezomib treatment, fixing protocol, Hoechst staining and secondary antibodies as above. The primary antibodies used were anti-phospho Aurora A Thr288 (Cell Signaling, #3079, 1:500) and anti-Histone H3 phospho S10 (Abcam, 14955, 1:2000). High-content imaging settings were the same as for the mislocalisation assay, but analysis was performed using the CellHealthProfiling v4 Bioapplication. Cells were identified in the Hoechst channel as above, and the resulting nuclear mask used for all subsequent measurements. Mitotic cells were selected from the images on the basis of phospho-histone H3 positivity, using a threshold 10 times above the image background. These selected cells were then measured for phospho-Aurora A staining intensity within a target area defined by expanding the nuclear mask by 8 pixels then collapsing it using a threshold for the phospho-Aurora channel, set to 3 times the background staining in this channel. Assay thresholds were calculated from the negative control wells of each plate as for the mislocalisation assay and ‘% dephosphorylated Aurora A T288’ scores were calculated for each well. EC_50_ values were calculated from four-parameter dose-response curves that were fitted using Prism GraphPad software (La Jolla, CA).

### Synthesis of 2-(3-bromophenyl)-8-fluoroquinoline-4-carboxylic acid (AurkinA)

7-fluoroisatin (165 mg, 1.00 mmol), 3-bromoacetophenone (0.16 mL, 1.2 mmol) and KOH (168 mg, 8.27 mmol) were dissolved in EtOH (2 mL). The reaction mixture was heated sealed tube in microwave for 1 h at 120 °C and the solvent was removed in under reduced pressure. The residue was dissolved in H_2_O and washed with equal volume of Et_2_O twice. The aqueous layer was cooled to 0 °C, acidified to pH 2 with HCl (conc.) and the precipitate was collected as a crude product which was subsequently recrystallised from 30% EtOH in H_2_O to yield the pure product (22 mg, 10%). ^**1**^**H NMR** (500 MHz; DMSO-d_6_): δ 14.18 (s, 1H), 8.58 (s, 1H), 8.51 (d, *J* = 0.8 Hz, 1H), 8.46–8.43 (m, 1H), 8.33 (d, *J* = 7.8 Hz, 1H), 7.76 (dt, *J* = 8.0, 1.0 Hz, 1H), 7.72 (t, *J* = 6.6 Hz, 2H), 7.56 (t, *J* = 7.9 Hz, 1H); ^**13**^**C NMR** (126 MHz; DMSO-d_6_): δ 167.2, 157.4 (d, *J* = 255.2 Hz), 154.4, 139.8, 138.4 (d, *J* = 11.6 Hz), 138.1, 133.0, 131.2, 129.8, 128.1, 126.4, 125.2, 122.5, 121.4, 120.1, 114.5 (d, *J* = 18.2 Hz); **IR** (solid) ν_max_ 3200–2650 (O-H_acid_, broad), 1697 (C=O_acid_, sharp), 1587 (C=C_aromatic_, sharp), cm^−1^; **HRMS** *(m/z)*: [M-H]^+^ calculated for C_16_H_10_BrFNO_2_, 345.9879; found 345.9903.

## Additional Information

**How to cite this article**: Janeček, M. *et al*. Allosteric modulation of AURKA kinase activity by a small-molecule inhibitor of its protein-protein interaction with TPX2. *Sci. Rep.* **6**, 28528; doi: 10.1038/srep28528 (2016).

## Supplementary Material

Supplementary Information
