# Supplementary material for: Allosteric modulation of AURKA kinase activity by a small-molecule inhibitor of its protein-protein interaction with TPX2
Source: Sci Rep. 2016 Jun 24;6:28528. doi: 10.1038/srep28528 (PMC4919790; doi:10.1038/srep28528)
Supplement: Supplementary Information [file srep28528-s1.pdf]

Supplementary Information

Allosteric modulation of AURKA kinase activity by a small-molecule inhibitor of its protein-protein interaction with TPX2

Matej Janeček, Maxim Rossmann, Pooja Sharma, Amy Emery, David J. Huggins, Simon Stockwell, Jamie E. Stokes, Yaw S. Tan, Estrella G. Almeida, Bryn Hardwick, Ana J. Narvaez, , Marko Hyvönen, David Spring, Grahame McKenzie, Ashok R. Venkitaraman

Table of Contents

Supplementary Information 1
Supplementary Figure 1: Development of the Fluorescence Anisotropy (FA) assay 2
Supplementary Figure 2: HTS hits retested on FA assay in presence of ATP-site blocking JNJ-7706621 4
Supplementary Table 1: SAR table of selected synthesised fragment analogues 6
Supplementary Figure 3: ITC validation of Hit 1 7
Supplementary Figure 4: ITC: association of AurkinA with AURKA 8
Supplementary Table 2: Crystallographic data 9
Supplementary Figure 5: Bromine anomalous electron density 13
Supplementary figure 6: Binding modes of AA29, AA30 and AurkinA 14
Supplementary Figure 7: B factors upon AurkinA binding 15
Supplementary Figure 8: R179-E239 crystal contact in $P6_122$ and $P4_12_12$ 16
Supplementary Figure 9: Homogeneous Time-Resolved Fluorescence (HTRF) assay optimisation 17
Supplementary Figure 10: Co-immunopreciptation of Aurora A and TPX2-mCherry 18
Supplementary Methods 19
Supplementary Information References 31

Supplementary Figure 1: Development of the Fluorescence Anisotropy (FA) assay

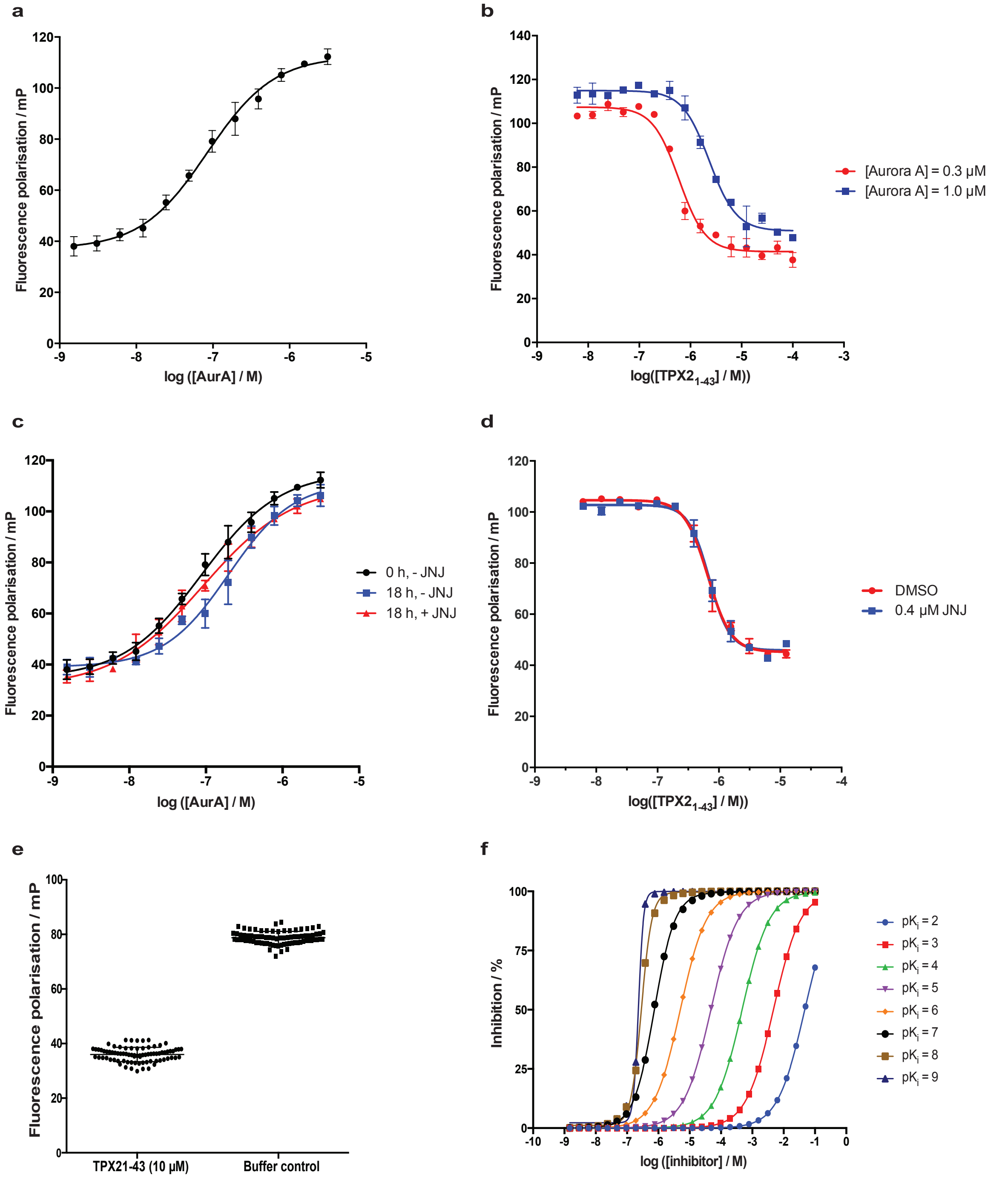

Supplementary Figure 1: The development of fluorescence anisotropy assay for high and low throughput screening. All anisotropy values were converted to equivalent polarisation for easier handling. Error bars denote ±SD, n=3. a) Titration of Aurora $A_{123\text{-}403}$ into TAMRA-$TPX2_{1\text{-}43}$ (12 nM) in PBS with DTT (300 μM). All subsequent studies used the same buffer conditions and TAMRA-$TPX2_{1\text{-}43}$ concentration. b) The comparison of the displacement of TAMRA-$TPX2_{1\text{-}43}$ by unlabelled $TPX2_{1\text{-}43}$ peptide from Aurora A at two different concentrations. [AurKA] = 0.3 μM was chosen for further studies in order to lower protein consumption and increase assay sensitivity. c) The effect of JNJ

(JNJ-7706621, excess, 0.4 µM) on stability of the AurKA protein over time. d) Comparison of Aurora A binding to TAMRA-$TPX2_{1-43}$ and its displacement by unlabelled $TPX2_{1-43}$ (positive control) in presence and absence of JNJ (0.4 µM). JNJ does not significantly alter the protein-peptide binding and was used in the assays after HTS to displace any ATP-site binders. e) The Z-Factor of the assay, z = 0.67, demonstrating a good assay. f) Calculated dynamic range of the FP assay[1], constrained by inhibitor solubility at the low-inhibitor-affinity end and TAMRA-$TPX2_{1-43}$ – Aurora A K_d at the high-inhibitor-affinity end. The ideal working dynamic range range was determined to be between mM and 100 nM.

Supplementary Figure 2: HTS hits retested on FA assay in presence of ATP-site blocking JNJ-7706621

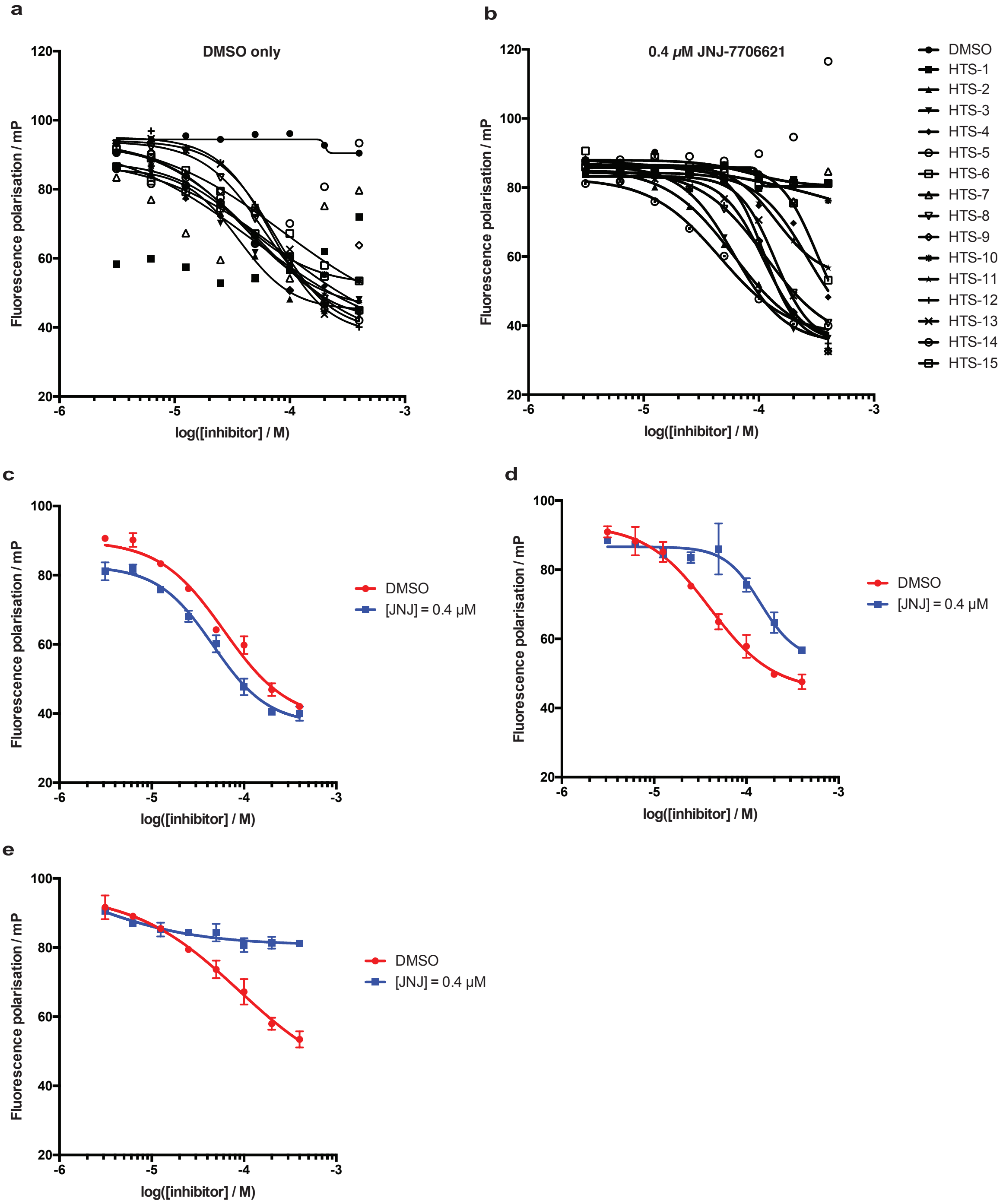

Figure 2: High throughput screening (HTS) hits retested on FA assay in presence of JNJ-7706621 (JNJ), an ATP-site blocker. The HTS hits were reordered and tested in FA assay. a) In presence of DMSO (control), 12 out of 15 reordered hit compounds show dose-dependent inhibition of the AURKA-TPX2 interaction. b) Excess of JNJ blocks ATP-site of AURKA and results in reduced IC_{50} values for most compounds. It also results in a more robust assay, and reduces amount of dose-independent inhibition (e.g HTS-1), likely due to the increased stability of AURKA protein. Detailed view of JNJ effect on HTS hits in FA assay: c) HTS-14 (**1**), inhibition of the AURKA-$TPX2_{1\text{-}43}$ interaction

unaffected by JNJ, d) HTS-13, inhibition of the interaction affected moderately (IC_{50} increased 3x), e) HTS-6, inhibition of the interaction ablated by addition of JNJ. Error bars denote ±SD, n=3.

Supplementary Table 1: SAR table of selected synthesised fragment analogues

Compound	(Iso)quinoline core	Aromatic group	Average IC_{50} / µM	Calculated K_i / µM
**3**		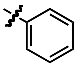	289	62.5
**24**		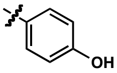	>500	>100
**26**		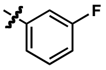	75.9	16.5
**AA30**	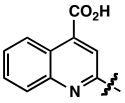	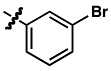	25.6	5.5
**33**		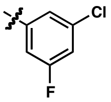	20.5	4.4
**32**		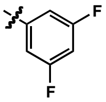	36.0	7.8
**34**		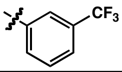	26.5	5.7
**31**		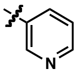	163	35.5
**25**	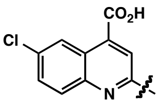	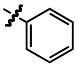	205	44.7
**27**		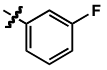	107	23.3
**AA29**		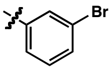	34.4	7.4
**28**	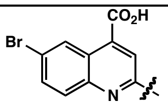	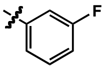	>500	>100
**AurkinA**	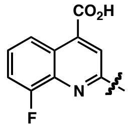	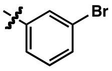	12.7	2.7
**50**	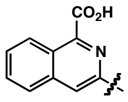	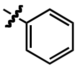	213	46.3
**51**		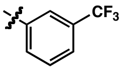	106	22.8

Supplementary Table 1: Selected synthesised fragment analogues and their activity in FA assay. K_i was calculated using free-concentration corrected Cheng-Prusov[2].

Supplementary Figure 3: ITC validation of Hit 1

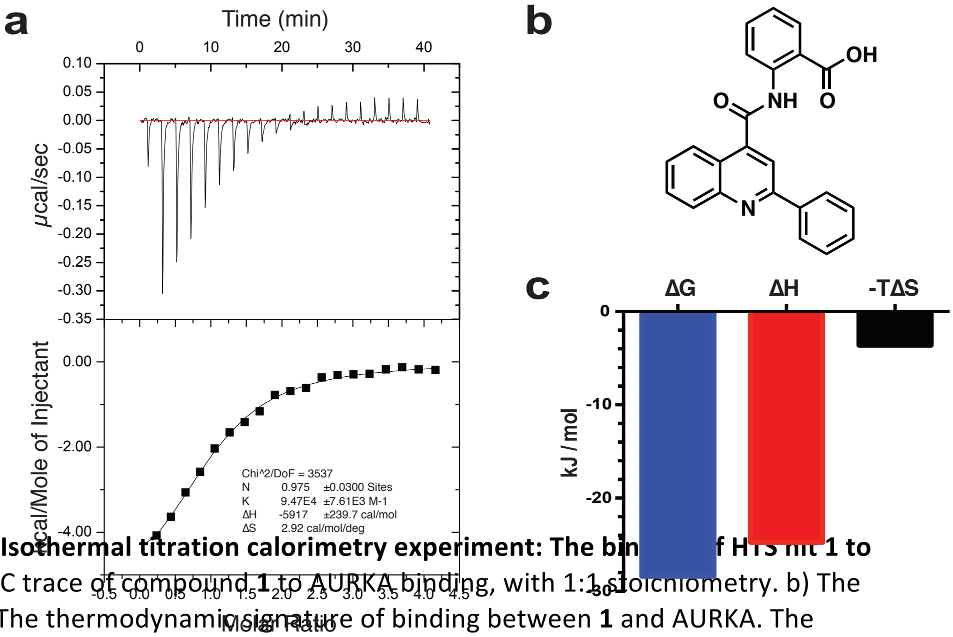

Supplementary figure 3: Isothermal titration calorimetry experiment: The bin[illegible]f HTS hit 1 to AURKA protein. a) The ITC trace of compound **1** to AURKA binding, with 1:1 stoichiometry. b) The structure of HTS hit **1**. c) The thermodynamic signature of binding between **1** and AURKA. The association was predominantly enthalpy-driven.

Supplementary Figure 4: ITC: association of AurkinA with AURKA

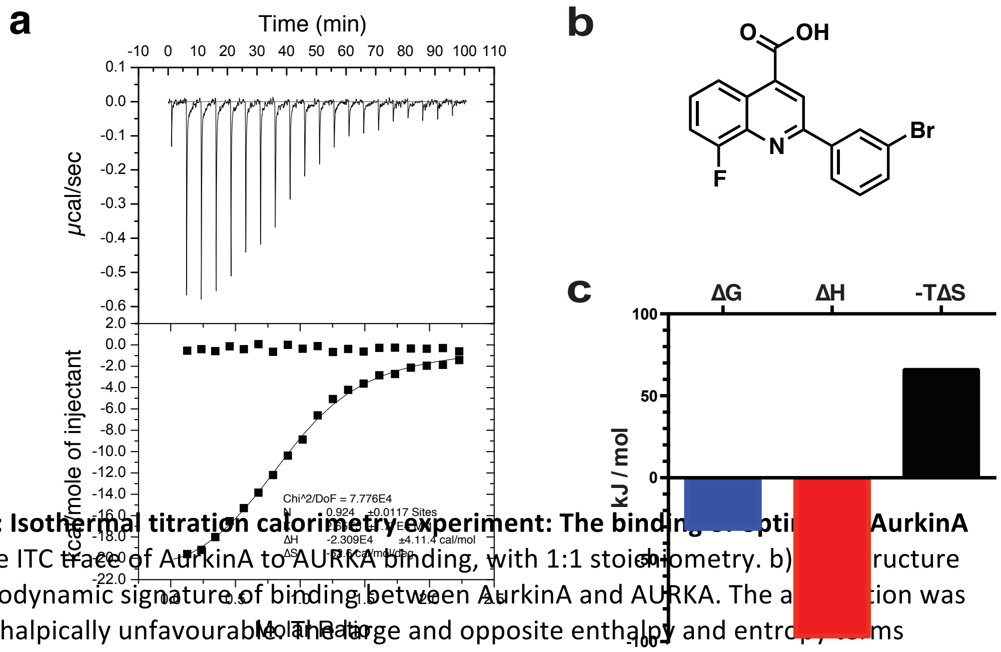

plementary Figure 4: Isothermal titration calorimetry experiment: The bindi[illegible] AurkinA .URKA protein. a) The ITC trace of AurkinA to AURKA binding, with 1:1 stoichiometry. b) [illegible]ructure urkinA. c) The thermodynamic signature of binding between AurkinA and AURKA. The a[illegible]tion was nalpy-driven, and enthalpically unfavourable. The large and opposite enthalpy and entro[illegible]ms gest an induction of significant conformation changes upon binding.

Supplementary Table 2: Crystallographic data

**Data collection**			
PDB ID	**5DNR**	**5DT3**	**5DT0**
Construct	AurA$_{GSMGS\text{-}126\text{-}391}$_ATP	AurA$_{G\text{-}126\text{-}390}$_ATP	AurA$_{G\text{-}126\text{-}390_T287A}$ _JNJ-7706621
X-Ray source	Diamond i04-1	Diamond i04-1	Diamond i03
Wavelength (Å)	0.9200	0.9200	0.92017
Resolution range (Å) (high res [illegible]	18.22-1.95 (2.020-1.950)	55.07-2.33 (2.410-2.330)	70.79-2.15 (2.230-2.150)
Space group	p41212	p6122	p6122
Unit cell (Å)	a=b=81.46 c=137.38	a=b=82.47 c=172.98	a=b=81.74 c=164.61
Total number of reflections	445823 (45384)	289984 (30232)	340085 (35018)
Number of unique reflections	34380 (3366)	15592 (1530)	18428 (1780)
Multiplicity (high res. shell)	13.0 (13.5)	18.6 (19.8)	18.5 (19.7)
Completeness (%) (high res. shell)	99.9 (99.9)	100 (100)	100 (100)
Mean I/sigma(I) (high res. shell)	27.7 (4.4)	31.1 (2.17)	18.9 (2.29)
R-merge (high res. shell)	0.056 (0.6837)	0.055 (1.612)	0.079 (1.118)
CC half ((high res. shell)	1.000 (0.931)	1.000 (0.749)	0.999 (0.824)
**Refinement**			
Resolution (Å) (high res. shell)	18.22-1.95 (2.00-1.95)	55.07-2.33 (2.49-2.33)	70.79-2.15 (2.28-2.15)
R-work (high res. shell)	0.206 (0.290)	0.210 (0.241)	0.211(0.215)
R-free (high res. shell)	0.226 (0.286)	0.243 (0.311)	0.225 (0.233)
Number of non-hydrogen atoms	2354	2285	2260
Protein	2196	2168	2131
Ligands	38	37	27
Waters	120	80	102
Protein residues	265	264	260
Root-mean-square deviation			
Bonds (Å)	0.015	0.015	0.014
Angles (°)	1.70	1.93	1.70
Average B-factor	39.4	73.56	66.79
Protein	40.70	73.59	66.93
Ligands	39.38	70.28	51.46
Solvent	45.30	74.16	67.85
Ramachandran favored (%)	97	97	97
Ramachandran outliers (%)	0	0.38	0.39
Clashscore	1.58	4.10	8.62

**Data collection**			
PDB ID	**5DRD**	**5DR9**	**5DR2**
Construct	$AurA_{G-126-390_T287A}$_ATP*	AA29_$AurA_{G-126-390_T287A}$_JNJ-7706621	AA30_$AurA_{G-126-390_T287A}$_ATP
X-Ray source	Diamond i03	Diamond i03	Diamond i03
Wavelength (Å)	0.92017	0.9184	0.9184
Resolution range (Å) (high res shell)	70.68-2.13 (2.206-2.130)	72.25-2.47 (2.558-2.470)	72.11-2.46 (2.548-2.460)
Space group	p6122	p6122	p6122
Unit cell (Å)	a=b=81.61 c=166.45	a=b=83.43 c=167.25	a=b=83.26 c=163.85
Total number of reflections	352124 (36035)	241410 (24764)	233520 (23677)
Number of unique reflections	19088 (1871)	13015 (1253)	12846 (1230)
Multiplicity (high res. shell)	18.4 (19.3)	18.5 (19.8)	18.2 (19.2)
Completeness (%) (high res. shell)	100 (100)	100 (100)	100 (100)
Mean I/sigma(I) (high res. shell)	17.94 (2.09)	27.3 (2.5)	18.76 (1.71)
R-merge (high res. shell)	0.090 (1.699)	0.060 (1.570)	0.089 (2.041)
CC half ((high res. shell)	0.999 (0.595)	0.999 (0.807)	0.999 (0.852)
**Refinement**			
Resolution (Å) (high res. shell)	70.68-2.13 (2.19-2.13)	72.25-2.47 (2.53-2.47)	72.11-2.46 (2.52-2.46)
R-work (high res. shell)	0.209 (0.394)	0.227 (0.211)	0.221 (0.239)
R-free (high res. shell)	0.262 (0.401)	0.262 (0.378)	0.285 (0.446)
Number of non-hydrogen atoms	2152	2174	2223
Protein	2120	2108	2162
Ligands	32	48	52
Waters	0	18	9
Protein residues	259	257	264
Root-mean-square deviation			
Bonds (Å)	0.017	0.017	0.017
Angles (°)	2.01	1.80	1.90
Average B-factor	59.70	93.40	80.77
Protein	58.80	93.63	82.78
Ligands	47.2	88.54	85.62
Solvent	-	79.32	69.54
Ramachandran favored (%)	96	95	93
Ramachandran outliers (%)	0	1.3	1.53
Clashscore	5.37	12.62	7.74

* -crystals were soaked with AA29, but no AA29 was found in the crystals structure

**Data collection**			
PDB ID	**5DR6**	**5DOS**	**5DPV**
Construct	AA30_AurA$_{G-126-390_T287A}$_JNJ-7706621	AurkinA_AurA$_{G-126-390_T287A}$_ATP	AurkinA_AurA$_{G-126-390_T287A}$_JNJ-7706621
X-Ray source	Diamond i03	Diamond i03	Diamond i03
Wavelength (Å)	0.9184	0.9184	0.9184
Resolution range (Å) (high res shell)	44.02-2.534 (2.624-2.534)	66.23-2.98 (3.087-2.980)	72.54-2.29 (2.372-2.290)
Space group	p6122	p6122	p6122
Unit cell (Å)	a=b=83.71 c=136.229	a=b=83.08 c=169.39	a=b=83.76 c=166.471
Total number of reflections	291643 (23058)	136661 (13546)	304281 (31470)
Number of unique reflections	12081 (1170)	7596 (735)	16267 (1579)
Multiplicity (high res. shell)	18.3 (19.7)	18.0 (18.4)	18.7 (19.9)
Completeness (%) (high res. shell)	100 (100)	100 (100)	100 (100)
Mean I/sigma(I) (high res. shell)	24.83 (2.86)	20.23 (2.52)	25.95 (2.33)
R-merge (high res. shell)	0.067 (1.063)	0.088 (1.193)	0.056 (1.446)
CC half ((high res. shell)	0.999 (0.895)	0.999 (0.901)	1.000 (0.845)
**Refinement**			
Resolution (Å) (high res. shell)	54.63-2.53 (2.60-2.53)	66.23-2.98 (3.33-2.98)	72.54-2.29 (2.44-2.28)
R-work (high res. shell)	0.221 (0.257)	0.201 (0.232)	0.225 (0.265)
R-free (high res. shell)	0.278 (0.362)	0.273 (0.363)	0.280 (0.265)
Number of non-hydrogen atoms	2177	2173	2214
Protein	2119	2120	2129
Ligands	47	53	48
Waters	11	0	37
Protein residues	257	259	257
Root-mean-square deviation			
Bonds (Å)	0.015	0.014	0.014
Angles (°)	1.74	1.82	1.74
Average B-factor	87.64	112.56	85.83
Protein	87.88	112.18	85.99
Ligands	81.57	127.76	81.89
Solvent	65.91	-	81.55
Ramachandran favored (%)	95	90	95
Ramachandran outliers (%)	0.39	0.78	1.2
Clashscore	10.46	6.74	6.24

**Data collection**		
PDB ID	**5DT4**	**5DN3**
Construct	AurkinA-AurA$_{G\text{-}126\text{-}390}$_ATP	AurkinA_AurA$_{GSMGS\text{-}126\text{-}391}$_ATP
X-Ray source	Diamond i02	Diamond i04-1
Wavelength (Å)	0.92017	0.9200
Resolution range (Å) (high res shell)	56.67-2.86 (2.930-2.860)	25.91-2.05 (2.123-2.050)
Space group	p6122	p41212
Unit cell (Å)	a=b=82.50 c=170.02	a=b=81.65 c=136.91
Total number of reflections	154948 (15279)	292874 (29439)
Number of unique reflections	8453 (810)	29721 (2910)
Multiplicity (high res. shell)	18.3 (18.9)	9.9 (10.1)
Completeness (%) (high res. shell)	100 (100)	99.8 (100)
Mean I/sigma(I) (high res. shell)	26.2 (1.7)	27.1 (3.9)
R-merge (high res. shell)	0.072 (2.188)	0.050 (0.633)
CC half ((high res. shell)	1.0000 (0.601)	1.000 (0.901)
**Refinement**		
Resolution (Å) (high res. shell)	54.70-2.86 (3.20-2.86)	25.91-2.05 (2.12-2.05)
R-work (high res. shell)	0.193 (0.253)	0.192 (0.219)
R-free (high res. shell)	0.256 (0.339)	0.203 (0.255)
Number of non-hydrogen atoms	2173	2405
Protein	2120	2200
Ligand	53	58
Waters	0	147
Protein residues	259	265
Root-mean-square deviation		
Bonds (Å)	0.014	0.015
Angles (°)	1.84	1.79
Average B-factor	112.4	45.62
Protein	111.2	44.71
Ligand	147.2	52.48
Solvent	-	56.45
Ramachandran favored (%)	93	96
Ramachandran outliers (%)	1.6	0
Clashscore	9.06	1.79

Supplementary Figure 5: Bromine anomalous electron density

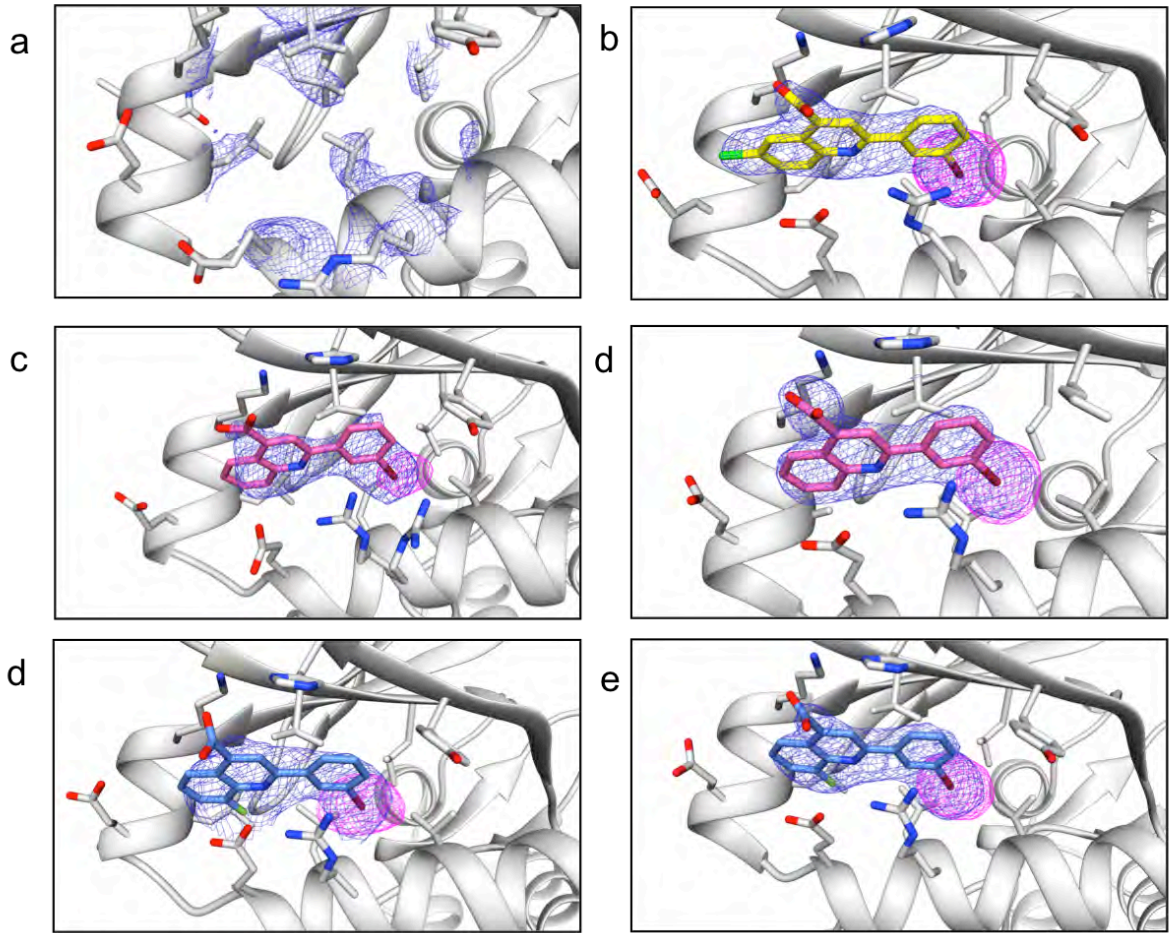

Supplementary Figure 5: AA29, AA30 and AurkinA binding mode in presence of Mg^{2+}-ATP (a, c, d) or JNJ-7706621 (b, d, e) in the ATP pocket. 2Fo-Fc map is countered at 1σ, anomalous map is countered at 5σ.

Supplementary figure 6: Binding modes of AA29, AA30 and AurkinA

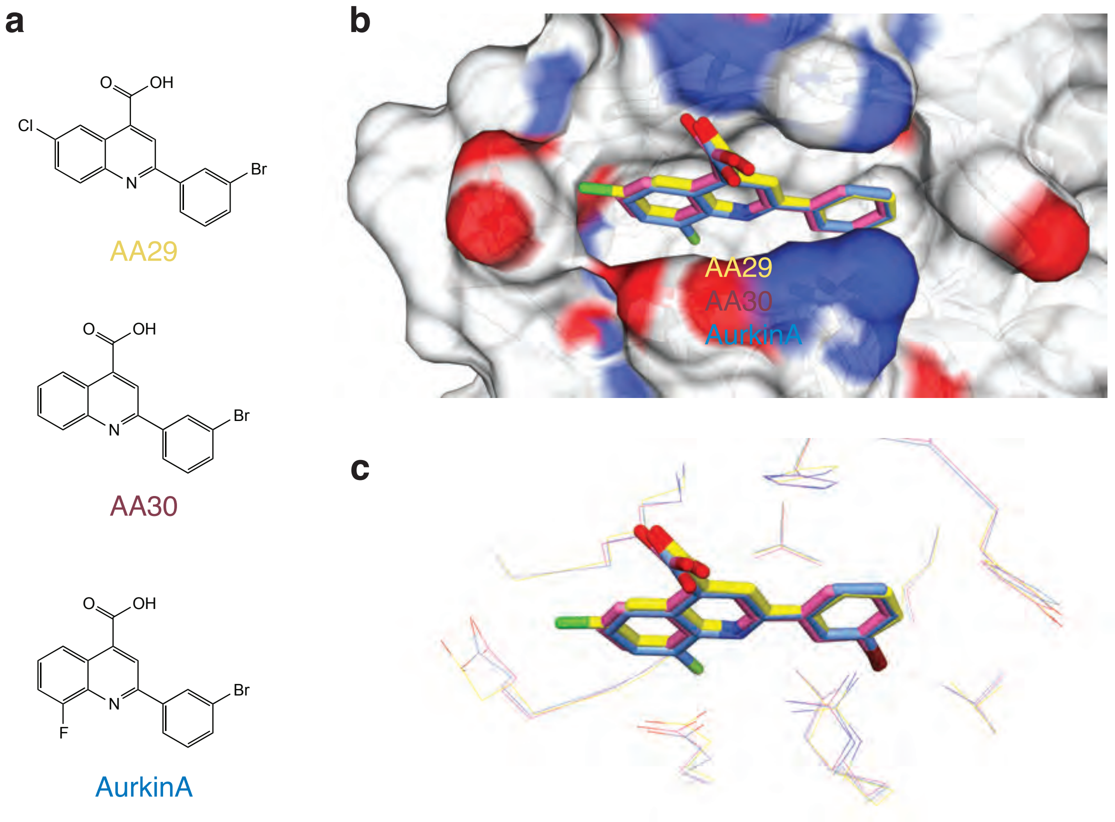

Supplementary Figure 6: (a) Structural formulas of AA29, AA30 and AurkinA**.** (b) Superimposition of the AA29 **(yellow)**, AA30 **(magenta)** and AurkinA **(blue)** binding mode in the presence of JNJ-7706621 in the ATP pocket. Binding pocket is represented as surface. (c) Superimposition of the AA29 **(yellow)**, AA30 **(magenta)** and AurkinA **(blue)** binding mode in the presence of JNJ-7706621 in the ATP pocket. Amino acids side chains in the binding pocket are represented as lines.

Supplementary Figure 7: B factors upon AurkinA binding

Construct	AurA$_{G\text{-}126\text{-}390_T287A}$	**AurkinA**_AurA$_{G\text{-}126\text{-}390_T287A}$
Ligand in the ATP side<br>Mg-ATP	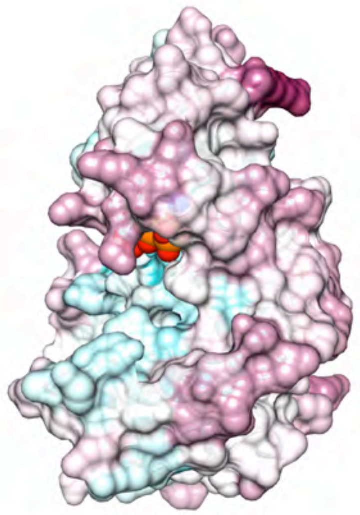	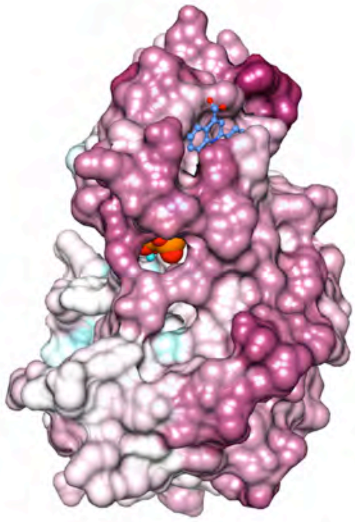
Ligand in the ATP side<br>JNJ-7706621	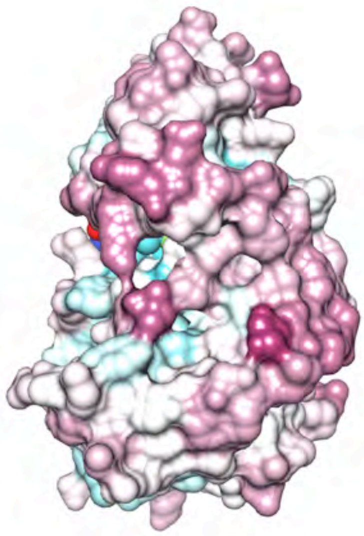	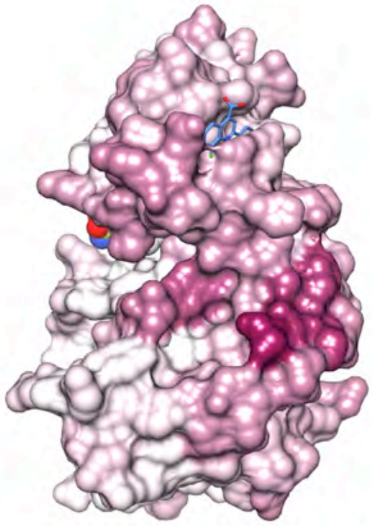

Supplementary Figure 7: B-factor changes upon AurkinA binding in presence of Mg^{2+}-ATP or JNJ-7706621 in the ATP pocket. B factor colouring was performed using PyMOL.

Supplementary Figure 8: R179-E239 crystal contact in $P6_122$ and $P4_12_12$

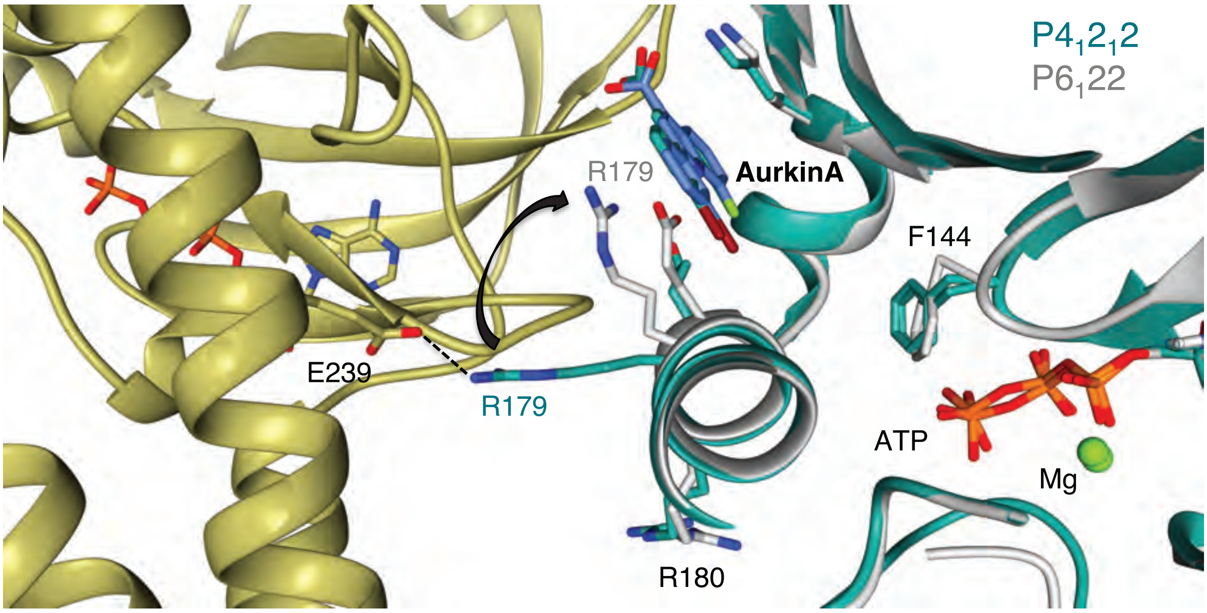

Supplementary Figure 8: R179-E239 crystal contact in space group $P6_122$ (grey) and $P4_12_12$ (green) upon AurkinA binding in presence of Mg-ATP. A salt bridge between R179 and E239 is present in the $P4_12_12$ crystals. In $P6_122$ crystals helix C rotates upon AurkinA binding resulting in the loss of the R179 - E239 crystal contact.

Supplementary Figure 9: Homogeneous Time-Resolved Fluorescence (HTRF) assay optimisation

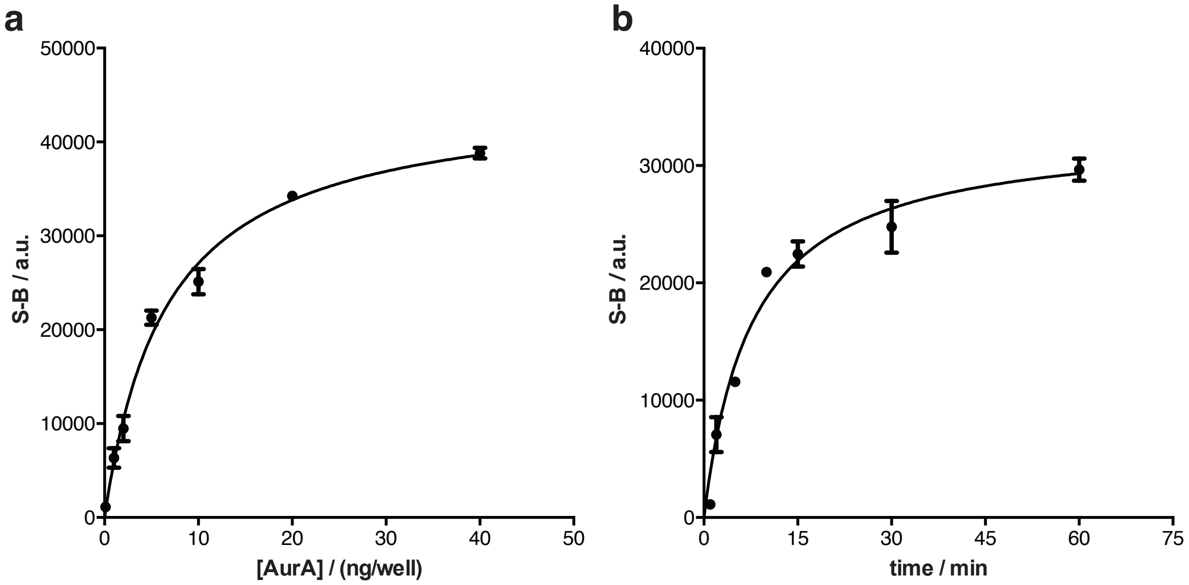

Supplementary Figure 9: HTRF assay kit (KinEASE, Cisbio) optimisation in 10 µL reaction volume, 20 µL detection volume. Phosphorylation of proprietary substrate was followed in the reaction. S-B denotes signal – background readout, and error bars denote standard deviation (n = 3) a) The titration of $AURKA_{126-390}$ in ATP (100 µM) and substrate (1 µM). 5 ng/well was determined to be optimal AURKA concentration for further studies. 30 min enzymatic step. b) The timecourse of AURKA (5 ng/mol) phosphorylation of the peptide substrate (1 µM) in ATP (100 µM). 10 min was the chosen time point for further reactions.

Supplementary Figure 10: Co-immunopreciptation of Aurora A and TPX2-mCherry

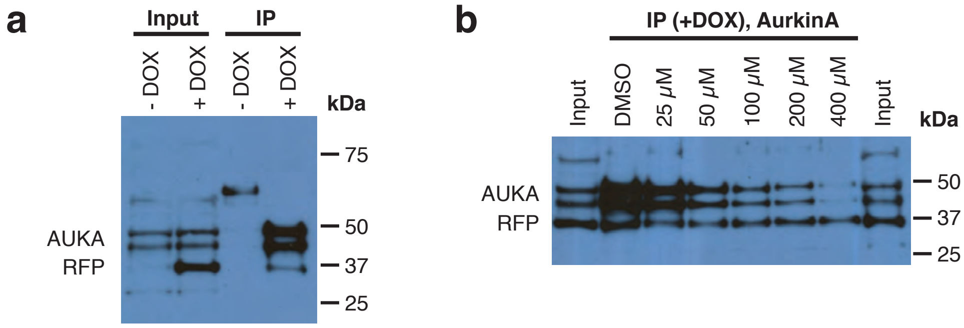

Supplementary Figure 10: Co-immunoprecipitation (CO-IP) of AURKA and mCherry-TPX2$_{1\text{-}43}$ with anti-RFP antibody from HeLa cell lysetes expressing mCherry-TPX2$_{1\text{-}43}$ fusion protein upon DOX-induction. (DOX = doxocycline). a) AURKA co-immunoprecipitated with mCherry-TPX2$_{1\text{-}43}$. (Input: 50 µg, IP: 500 µg of cell lysate). b) AurkinA dose-dependently inhibited the co-immunoprecipitation of AURKA with RFP in HeLa cells expressing mCherry-TPX2$_{1\text{-}43}$ in presence of DOX. Cell lysates (500 µg) were incubated with AurkinA for 2h. (Input: 50 / 25 µg)

Supplementary Methods

Preparation of human Aurora-A Kinase Expression Plasmids

Human Aurora-A kinase was produced using λPP co-expression method described for mouse Aurora-A[3]. Enterbacterial phage lambda phosphatase (λPP) gene (GenBank ID: 270346) was amplified by PCR using cloning primers 1 and 2 that included *Xho*I and *Hind*III restriction sites as well as the Shine-Dalgarno ribosome binding sequence. Digested and purified PCR product was ligated into pBAT4 and pHAT4 expression plasmids[4] linearized with *Xho*I and *Hind*III restriction endonucleases resulting in plasmids pBAT4-λPP and pHAT4-λPP, respectively.

Human Aurora-A kinase gene fragment encoding residues 126-390 was amplified by PCR using cloning primers 3 and 4, digested with *Nco*I and *Xho*I restriction endonucleases and ligated into the *Nco*I and *Xho*I linearised pBAT4-λPP plasmid upstream the λPP gene resulting in Aurora-A expression construct termed $AurA_{G\text{-}126\text{-}390}$.

$AurA_{G\text{-}126\text{-}390}$ T287A mutant was generated by PCR overlap extension method using cloning primers 3 and 4 and mutation primers 5 and 6.

Another Aurora-A kinase expression construct termed $AurA_{GSMGS\text{-}126\text{-}391}$ was cloned into pBAT4-λPP vector using cloning primers 7 and 8. $AurA_{GSMGS\text{-}126\text{-}391}$ expression construct encoded human Aurora-A kinase residues 126-391 and carried a TEV-cleavable N-terminal 6xHis-tag.

The Aurora-A kinase expression construct used for FA assay termed $AurA_{123\text{-}403}$, was produced differently. The Human Aurora-A kinase gene fragment encoding residues 123-403 was amplified by PCR using cloning primers 9 and 10. It was then digested by *ClaI* and *XhoI* restriction endonucleases and ligated into the *ClaI* and *XhoI* linearised pBAD-HisA vector (Invitrogen), prepared in SCS110 E.coli to prevent methylation of *ClaI* site. $AurA_{123\text{-}403}$ expression construct encoded an N-terminal region carrying 6xHis-tag as well as EK recognition site (sequence: MGGSHHHHHHGMASMTGGQQM GRDLYDDDDKDR) in addition to Aurora-A kinase residues 123-403.

Primer	Sequence
1	5'-GATCACTCGAGCAATTTCACACAGGAAACAGTATTCATGCGCTATTACGAAAAAATTGATGGCAGC-3'
2	5'-CTAAGCTTGTCGACTCATGCGCCTTCTCCCTGTACCTGAATCAATG-3'
3	5'-AGTTACCATGGGTAGGCAGTGGGCTTTGGAAGACTTTGAAATTG-3'
4	5'-GACGTTCTCGAGTTAATGATGATGATGATGATGTGGTTTTGATGAATTTGCTGTGATCC-3'

5	5’-GCTCCATCTTCCAGGAGGGCTACTCTCTGTGGCACCCTGGAC-3’
6	5’-GTCCAGGGTGCCACAGAGAGTAGCCCTCCTGGAAGATGGAGC-3’.
7	5’-ATGGATCCATGGGATCTAAGAGGCAGTGGGCTTTGGAAGACTTTG-3’
8	5’-TGAGCTCGAGCTATGATGGTTTTGATGAATTTGCTGTGATCC-3’.
9	5’-GCCGATCGATCAAAAAAGAGGCAGTG-3’
10	5’-AGGATCACTCGAGCTAAGAC-3’

Generation of stable cell line, maintenance and selection

HeLa FlpIn TREx cell line (a kind gift from Professor Stephen Taylor, University of Manchester) was used to generate stable cell line. The cells were maintained in DMEM medium (with GlutaMax, Invitrogen) with 10% FCS and Zeocin (50 µg ml^{-1}) and blasticidin (4 µg ml^{-1}).

The mCherry-$TPX2_{1\text{-}43}$ construct was prepared by cloning mCherry and $TPX2_{1\text{-}43}$ sequences into pCDNA3.1 (-) vector. The mCherry gene was amplified by PCR using primers 11 and 12, and cloned into pCDNA3.1 (-) vector by *XbaI* and *XhoI* restriction endonucleases. The $TPX2_{1\text{-}43}$ gene fragment was constructed by annealing designed oligomers (13, 14, 15 and 16) followed by 5’end phosphorylation by T4 polynucleotide kinase. The $TPX2_{1\text{-}43}$ sequence was cloned into the pCDNA3.1 (-) vector at *EcoRI* and *BamHI* sites.

Primer	Sequence
11	5’-ATGC TCTAGA ATG GTG AGC AAG GGC GAG GAG GAT-3’
12	5’-ATGC CTCGAG CTT GTA CAG CTC GTC CAT GCC GCC GGT 3’
13	5’-AATTC ATG TCC CAG GTG AAG AGC TCC TAC TCC TAC GAC GCC CCC TCC GAC TTC ATC AAC TTC TCC TCC CTG GAC GAC-3’
14	5’-GAG GGC GAC ACC CAG AAC ATC GAC TCC TGG TTC GAG GAG AAG GCC AAC CTG GAG AAC TGA G-3’
15	5’-GATCC TCA GTT CTC CAG GTT GGC CTT CTC CTC GAA CCA GGA GTC GAT GTT CTG GGT GTC GCC CTC GTC GTC CAG GGA GGA-3’
16	5’-GAA GTT GAT GAA GTC GGA GGG GGC GTC GTA GGA GTA GGA GCT CTT CAC CTG GGA CAT G-3’

mCherry-$TPX2_{1\text{-}43}$ was sub-cloned from the previously generated pCDNA3.1 (-) construct (see above) into pcDNA5/FRT/TO vector at *PmeI* site. The clones generated were tested by colony PCR and the

directionality of insert was established using *EcoRI* / *XbaI* restriction digestion. A construct with desired orientation were verified by sequencing and used for generating the stable cell line.

The mCherry-TPX2 construct was co-transfected with pOG44 (Invitrogen) using JETPrime reagent following manufacturer's instructions. Hygromycin (200 µg ml^{-1}) and blasticidin resistant cells were selected, pooled and expanded. The cells were always maintained on antibiotic containing medium.

Pooled clones were sorted by a cell sorter (BD Biosciences) into 96-well plate with one cell per well. The medium was changed regularly and when cells had expanded these were replica plated and tested for the expression of mCherry-TPX2 by high content microscopy as described below. A clone with high expression and low background was selected, expanded and used for further experiments.

Preparation of Supplementary Video

The video depicts the transition between the Aurora A crystals structure containing ATP ($AurA_{G\text{-}126\text{-}390}$_T287A_ATP (5DRD)) and crystals structure of Aurora A with bound ATP and AurkinA (AurkinA_$AurA_{G\text{-}126\text{-}390}$_T287A_ATP (5DOS)). The video was prepared by 'morphing' from the *apo* structure to the AurkinA complex and back, using UCSF chimera package[5].

Chemical Synthesis of AURKA-TPX2 inhibitor analogues

General procedure: Pfitzinger reaction

According to the procedure described by Giardina *et al.*[6], isatin (1.0 eq), acetophenone (1.2 eq) and KOH (3.0 eq) were dissolved in EtOH. The reaction mixture was heated under reflux for 48-72 h and the solvent was removed under reduced pressure. The residue was dissolved in H_2O and washed with equal volume of Et_2O twice. The aqueous layer was cooled to 0 °C, acidified to pH 2 with (conc.) HCl and the precipitate was collected as a crude product, which was then triturated or recrystallised.

methyl 2-(2-phenylquinoline-4-carboxamido)benzoate

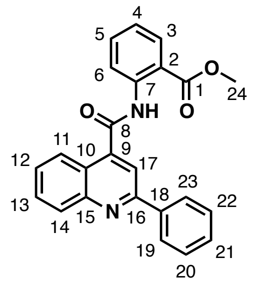

Methyl anthranilate (0.30 mL, 2.41 mmol), cinchophen (500 mg, 2.01 mmol) and DIPEA (0.70 mL, 4.01 mmol) were added to oven-dried round-bottomed flask containing dry EtOAc (10 mL). The mixture was cooled to 0 °C and T3P (50% in EtOAc, 1.8 mL, 3.01 mmol) was added dropwise and the reaction mixture was stirred at 0 °C for 30 min. The mixture was allowed to warm up to RT and after overnight stirring, the reaction was quenched by addition of H_2O. The mixture was diluted with EtOAc and the organic layer was washed with (sat.) $NaHCO_3$, brine, dried ($MgSO_4$), filtered and the solvent was removed under reduced pressure. The crude product was purified by flash column

chromatography (30% EtOAc, 70% Petroleum ether 40-60) to yield the product as yellow solid (472 mg, 62 %). **R$_f$** (30% EtOAc, 70% Pet. Ether 40-60) = 0.33; **IR** (solid) ν_{max} 1696 (C=O$_{ester}$, sharp), 1683 (C=O$_{amide}$, broad), cm^{-1}; **^{1}H NMR** (500 MHz; DMSO-d$_6$): δ 8.44 (s, 1H, C17-H), 8.35 (d, *J* = 7.3 Hz, 2H, C19-H, C23-H), 8.33 (d, *J* = 8.7 Hz, 1H, C^{Ar}-H), 8.25 (d, *J* = 8.2 Hz, 1H, C6-H), 8.20 (d, *J* = 8.4 Hz, 1H, C^{Ar}-H), 7.96 (d, *J* = 6.6 Hz, 1H, C3-H), 7.88 (t, *J* = 7.3 Hz, 1H, C^{Ar}-H), 7.76-7.69 (m, 2H, C5-H, C^{Ar}-H), 7.61 (t, *J* = 7.3 Hz, 2§H, C20-H, C22-H), 7.56 (t, *J* = 7.2 Hz, 1H, C21-H), 7.36 (t, *J* = 7.4 Hz, 1H, C4-H), 3.82 (s, 3H, C24-H$_3$); **^{13}C NMR** (126 MHz; DMSO-d$_6$): δ 167.4 (C1), 165.2 (C8), 155.9 (Cq^{Ar}), 148.0 (Cq^{Ar}), 142.5 (Cq^{Ar}), 138.2 (Cq^{Ar}), 138.0 (Cq^{Ar}), 133.6 (C5), 130.5 (C3), 130.4 (C13), 130.1 (C21), 129.6 (C14), 129.0, (C20, C22) 127.6 (C12), 127.3 (C19, C23), 125.1 (C11), 124.7 (C4), 123.0 (C6), 122.9 (Cq^{Ar}), 120.9 (Cq^{Ar}), 116.7 (C17), 52.5 (C24); **HRMS** *m/z* (ESI+) $C_{24}H_{19}N_2O_3$ calculated [M+H]$^+$ 383.1396, found 383.1447.

2-(2-phenylquinoline-4-carboxamido)benzoic acid (1)

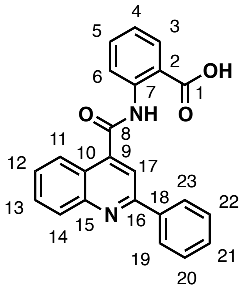

Using the general procedure, the methyl ester **11** (100 mg, 0.26 mmol) was hydrolysed to yield pure product as white solid (86 mg, 84%). **R$_f$** (100% EtOAc) = 0.15; **IR** (solid) ν_{max} 3150-2750 (O-H$_{acid}$, broad), 1680 (C=O$_{acid}$, sharp), 1660 (C=O$_{amide}$, broad), cm^{-1}; **^{1}H NMR** (500 MHz; DMSO-d$_6$): δ 13.72 (s, 1H, COO-H), 12.14 (s, 1H, -NH), 8.61 (d, *J* = 8.2 Hz, 1H, C6-H), 8.49 (s, 1H, C17-H), 8.38 (dd, *J* = 8.4, 0.8 Hz, 1H, C11-H), 8.35 (d, *J* = 7.0 Hz, 2H, C23-H, C19-H), 8.19 (dd, *J* = 8.5, 0.6 Hz, 1H, C14-H), 8.06 (dd, *J* = 7.9, 1.5 Hz, 1H, C3-H), 7.88 (ddd, *J* = 8.4, 6.9, 1.4 Hz, 1H, C13-H), 7.71 (m, 2H, C5-H, C12-H), 7.62-7.54 (m, 3H, C20-H, C21-H, C22-H), 7.30 (td, *J* = 7.6, 0.9 Hz, 1H, C4-H); **^{13}C NMR** (126 MHz; DMSO-d$_6$): δ 169.4 (C1), 165.0 (C8) 156.0 (Cq^{Ar}), 148.2 (Cq^{Ar}), 142.6 (Cq^{Ar}), 139.9 (Cq^{Ar}), 138.0 (Cq^{Ar}), 133.7 (C5), 131.1 (C3), 130.5 (C13), 130.1 (C21), 129.7 (C14), 129.0 (C20, C22), 127.6 (C12), 127.3 (C19, C23), 125.2 (C11), 123.9 (C4), 123.0 (Cq^{Ar}), 121.0 (C6), 119.3 (Cq^{Ar}), 116.6 (C17); **HRMS** *m/z* (ESI+) $C_{23}H_{17}N_2O_3$ calculated [M+H]$^+$ 369.1239, found 369.1263.

2-(4-hydroxyphenyl)quinoline-4-carboxylic acid (24)

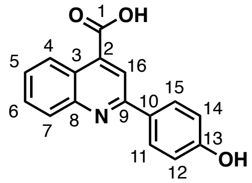

Using the general procedure, isatin (1.00 g, 6.8 mmol), 4-hydroxyacetophenone (1.111 g, 8.16 mmol) and KOH (1.144 g, 20.4 mmol) were combined yielding the crude product, which was subsequently recrystallised from EtOH to yield the pure product (125 mg, 13%). **IR** (solid) ν_{max} 3400-2700 (O-H$_{acid}$, broad), 1633 (C=O$_{acid}$, sharp), 1611 (C-O$_{aromatic}$, sharp), 1583 (C=C$_{aromatic}$, sharp), cm^{-1}; **^{1}H NMR** (500 MHz; DMSO-d$_6$): δ 13.91 (s, 1H, COO-H), 9.93 (s, 1H, O-H), 8.60 (dd, *J* = 8.5, 0.8 Hz, 1H, C7-H), 8.37 (s,

1H, C16-H), 8.18-8.15 (m, 2H, C11-H, C15-H), 8.09 (ddd, *J* = 8.5, 1.2, 0.6 Hz, 1H, C4-H), 7.81 (ddd, *J* = 8.4, 6.9, 1.5 Hz, 1H, C5-H), 7.64 (ddd, *J* = 8.4, 6.9, 1.4 Hz, 1H, C6-H), 6.95-6.92 (m, 2H, C12-H, C14-H); **^{13}C NMR** (126 MHz; DMSO-d$_6$): δ 168.2 (C1), 159.9 (C13), 156.2 (Cq^{Ar}), 148.86 (Cq^{Ar}), 137.7 (Cq^{Ar}), 130.5 (C5), 129.9 (C4), 129.2 (C11, C15), 127.5 (C6), 125.8 (C7), 123.4 (Cq^{Ar}), 119.0 (C16)), 116.2 (C12, C14); **HRMS** *m/z* (ESI+) $C_{16}H_{12}NO_3$ calculated $[M+H]^+$ 266.0817, found 266.0839.

6-chloro-2-phenylquinoline-4-carboxylic acid (25)

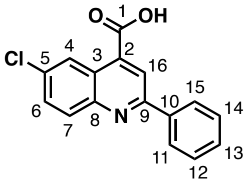

Using the general procedure, 5-chloroisatin (1.00 g, 5.51 mmol), acetophenone (0.77 g, 6.61 mmol) and KOH (0.93 g, 16.53 mmol) were combined to yield the crude product, which was subsequently recrystallised from EtOH to yield the pure product (190 mg, 13%). **IR** (solid) ν_{max} 3200-2750 (O-H$_{acid}$, broad), 1718 (C=O$_{acid}$, sharp), 1588 (C=C$_{aromatic}$, sharp), cm^{-1}; **^{1}H NMR** (500 MHz; DMSO-d$_6$): δ 14.18 (s, 1H (COO-H), 8.78 (d, *J* = 2.4 Hz, 1H, C4-H), 8.54 (s, 1H, C16-H), 8.28 (dq, *J* = 6.3, 2.0 Hz, 2H, C11-H, C15-H), 8.18 (dd, *J* = 9.0, 0.4 Hz, 1H), C7-H), 7.87 (dd, *J* = 9.0, 2.4 Hz, 1H, C6-H), 7.60-7.53 (m, 3H, C12-H, C13-H, C14-H); **^{13}C NMR** (126 MHz; DMSO-d$_6$): δ 167.5 (C1), 156.8 (Cq^{Ar}), 147.4 (Cq^{Ar}), 137.9 (Cq^{Ar}), 136.6 (Cq^{Ar}), 132.9 (Cq^{Ar}), 132.3 (C7), 131.2 (C6), 130.7 (C13), 129.5 (C12, C14), 127.7 (C11, C15), 124.79 (C4), 124.74 (Cq^{Ar}), 121.0 (C16); **HRMS** *m/z* (ESI+) $C_{16}H_{11}ClNO_2$ calculated $[M+H]^+$ 284.0478, found 284.0494.

2-(3-fluorophenyl)quinoline-4-carboxylic acid (26)

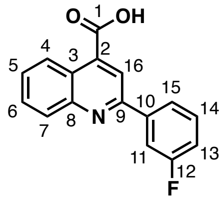

Using the general procedure, isatin (500 mg, 3.4 mmol), 3-fluoroacetophenone (0.50 mL, 4.08 mmol) and KOH (572 mg, 10.2 mmol) were combined yielding the crude product, which was subsequently recrystallised from EtOH to yield the pure product (322 mg, 35%). **IR** (solid) ν_{max} 3200-2700 (O-H$_{acid}$, broad), 1698 (C=O$_{acid}$, sharp), 1588 (C=C$_{aromatic}$, sharp), cm^{-1}; **^{1}H NMR** (500 MHz; DMSO-d$_6$): δ 14.04 (s, 1H, COO-H), 8.64 (dt, *J* = 8.5, 0.6 Hz, 1H, C7-H), 8.49 (s, 1H, C16-H), 8.19 (ddd, *J* = 8.5, 1.3, 0.6 Hz, 1H, C4-H), 8.16 (ddd, *J* = 7.8, 1.5, 0.9 Hz, 1H, C15-H), 8.12 (ddd, *J* = 10.6, 2.5, 1.6 Hz, 1H, C11-H), 7.87 (ddd, *J* = 8.4, 6.9, 1.5 Hz, 1H, C5-H), 7.73 (ddd, *J* = 8.4, 6.9, 1.4 Hz, 1H, C6-H), 7.62 (td, *J* = 8.0, 6.1 Hz, 1H, C14-H), 7.38 (tdd, *J* = 8.5, 2.6, 0.7 Hz, 1H, C13-H); **^{13}C NMR** (126 MHz; DMSO-d$_6$): δ 167.5, 162.7 (d, *J* = 243.9 Hz, C12), 154.4 (d, *J* = 2.3 Hz, Cq^{Ar}), 148.2 (Cq^{Ar}), 140.4 (Cq^{Ar}), 138.0 (Cq^{Ar}), 131.0 (d, *J* = 8.3 Hz, C12), 130.4 (C4), 129.9 (C5), 128.1 (C6), 125.4 (C7), 123.6 (Cq^{Ar}), 123.3 (d, *J* = 2.1 Hz, C15), 119.1 (C16), 116.8 (d, *J* = 20.1 Hz, C13), 113.8 (d, *J* = 22.7 Hz, C11); **HRMS** *m/z* (ESI+) $C_{16}H_{11}FNO_2$ calculated $[M+H]^+$ 268.0774, found 268.0789.

6-chloro-2-(3-fluorophenyl)quinoline-4-carboxylic acid (27)

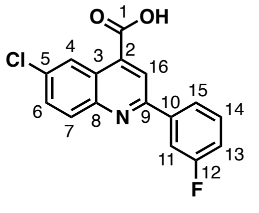

Using the general procedure, 5-chloroisatin (500 mg, 2.75 mmol), 3-fluoroacetophenone (0.42 mL, 3.44 mmol) and KOH (464 mg, 8.27 mmol) were combined yielding the crude product, which was subsequently recrystallised from EtOH to yield the pure product (65 mg, 8%). **IR** (solid) ν_{max} 3250-2700 (O-H_{acid}, broad), 1720 (C=O_{acid}, sharp), 1587 (C=$C_{aromatic}$, sharp), cm^{-1}; **^{1}H NMR** (500 MHz; DMSO-d_6): δ 14.19 (s, 1H, COO-H), 8.76 (d, *J* = 2.3 Hz, 1H, C4-H), 8.55 (s, 1H, C16-H)), 8.17 (dd, *J* = 9.0, 0.5 Hz, 1H, C7-H), 8.13 (ddd, *J* = 7.8, 1.6, 0.9 Hz, 1H, C15-H), 8.09 (ddd, *J* = 10.6, 2.5, 1.6 Hz, 1H, C11-H), 7.87 (dd, *J* = 9.0, 2.4 Hz, 1H, C6-H), 7.61 (td, *J* = 8.0, 6.1 Hz, 1H, C14-H), 7.38 (tdd, *J* = 8.5, 2.6, 0.7 Hz, 1H, C13-H); **^{13}C NMR** (126 MHz; DMSO-d_6): δ 167.0 (C1), 162.8 (d, *J* = 243.6 Hz, C12), 154.9 (d, *J* = 3.0 Hz, Cq^{Ar}), 146.8 (Cq^{Ar}), 140.0 (d, *J* = 7.6 Hz, Cq^{Ar}), 136.4 (Cq^{Ar}), 132.8 (Cq^{Ar}), 131.9 (C7), 131.1 (d, *J* = 7.9 Hz, C14), 130.8 (C6), 124.48 (Cq^{Ar}), 124.33 (C4), 123.4 (d, *J* = 2.4 Hz, C15), 120.6 (C16), 117.0 (d, *J* = 21.2 Hz, C13), 113.8 (d, *J* = 22.9 Hz, C11); **HRMS** *m/z* (ESI+) $C_{16}H_{10}ClFNO_2$ calculated $[M+H]^+$ 302.0384, found 302.0396.

6-bromo-2-(3-fluorophenyl)quinoline-4-carboxylic acid (28)

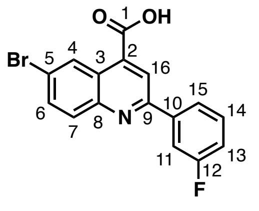

Using the general procedure, 5-bromoisatin (500 mg, 2.2 mmol), 3-fluoroacetophenone (0.33 mL, 2.7 mmol) and KOH (370 mg, 6.6 mmol) were combined yielding the crude product, which was subsequently recrystallised from 80 % EtOH in H_2O to yield the pure product (35 mg, 5%). **IR** (solid) ν_{max} 3200-2850 (O-H_{acid}, broad), 1699 (C=O_{acid}, sharp), 1587 (C=$C_{aromatic}$, sharp), cm^{-1}; **^{1}H NMR** (500 MHz; DMSO-d_6): δ 14.22 (s, 1H COO-H), 8.95 (d, *J* = 2.2 Hz, 1H, C4-H), 8.58 (s, 1H, C16-H), 8.18-8.12 (m, 3H, C7, C11, C15), 8.02 (dd, *J* = 9.0, 2.3 Hz, 1H, C6), 7.64 (td, *J* = 8.0, 6.1 Hz, 1H, C14), 7.41 (tdd, *J* = 8.5, 2.6, 0.7 Hz, 1H, C13); **^{13}C NMR** (126 MHz; DMSO-d_6): δ 167.0 (C1), 162.7 (d, *J* = 244.1 Hz, C12), 155.1 (Cq^{Ar}), 147.0 (Cq^{Ar}), 140.0 (Cq^{Ar}), 136.4 (Cq^{Ar}), 133.5 (C6), 132.0 (C7), 131.1 (d, *J* = 8.3 Hz, C14), 127.6 (C4), 125.0 (Cq^{Ar}), 123.4 (d, *J* = 1.9 Hz, C15), 121.6 (Cq^{Ar}), 120.5 (C16), 117.1 (d, *J* = 21.2 Hz, C13), 113.9 (d, *J* = 22.8 Hz, C11); **HRMS** *m/z* (ESI+) $C_{16}H_{10}BrFNO_2$ calculated $[M+H]^+$ 345.9879, found 345.9898.

2-(3-bromophenyl)-6-chloroquinoline-4-carboxylic acid (AA29)

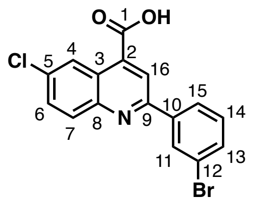

Using the general procedure, 5-chloroisatin (500 mg, 2.75 mmol), 3-bromoacetophenone (0.46 mL, 3.45 mmol) and KOH (464 mg, 8.27 mmol) were combined yielding the crude product. The crude product was recrystallised from EtOH to yield the pure product (92 mg, 8%). **IR** (solid) ν_{max} 3200-2700 (O-H_{acid}, broad), 1719 (C=O_{acid}, sharp), 1587 (C=$C_{aromatic}$, sharp), cm^{-1}; **^{1}H NMR** (500 MHz; DMSO-d_6): δ 14.18 (s, 1H, COO-H), 8.74 (d, *J* = 2.4 Hz, 1H, C4-H), 8.52 (s, 1H, C16-H), 8.44 (t, *J* = 1.8 Hz, 1H, C11-H), 8.25 (ddd, *J* = 7.9, 1.7, 1.0 Hz, 1H, C15-H), 8.16 (d, *J* = 9.0 Hz, 1H, C7-H), 7.85 (dd, *J* = 9.0, 2.4 Hz, 1H, C6-H), 7.72 (ddd, *J* = 7.9, 2.0, 0.9 Hz, 1H, C13-H), 7.52 (t, *J* = 7.9 Hz, 1H, C14-H); **^{13}C NMR** (126 MHz; DMSO-d_6): δ 166.9 (C1), 154.6 (Cq^{Ar}), 146.8 (Cq^{Ar}), 139.7 (Cq^{Ar}), 136.4 (Cq^{Ar}), 132.86 (C13), 132.81 (Cq^{Ar}), 131.9 (C7), 131.1 (C14), 130.8 (C6), 129.7 (C11), 126.3 (C15), 124.5 (Cq^{Ar}), 124.32 (C4), 122.5 (Cq^{Ar}), 120.5 (C16); **HRMS** *m/z* (ESI+) $C_{16}H_{10}BrClNO_2$ calculated $[M+H]^+$ 361.9583, found 361.9604.

2-(3-bromophenyl)quinoline-4-carboxylic acid (AA30)

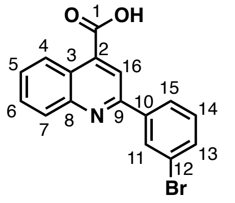

Using the general procedure, isatin (500 mg, 3.4 mmol), 3-bromoacetophenone (0.46 mL, 3.45 mmol) and KOH (506 mg, 9.0 mmol) were combined yielding the crude product, which was subsequently recrystallised from EtOH to yield the pure product (425 mg, 38%). **IR** (solid) ν_{max} 3200-2600 (O-H_{acid}, broad), 1715 (C=O_{acid}, sharp), 1593 (C=$C_{aromatic}$, sharp), cm^{-1}; **^{1}H NMR** (500 MHz; DMSO-d_6): δ 14.05 (s, 1H, COO-H), 8.64 (dt, *J* = 8.5, 0.7 Hz, 1H, C7), 8.50 (t, *J* = 1.8 Hz, 1H, C11), 8.49 (s, 1H, C16), 8.30 (ddd, *J* = 7.9, 1.7, 1.0 Hz, 1H, C15), 8.20 (ddd, *J* = 8.5, 1.3, 0.6 Hz, 1H, C4), 7.87 (ddd, *J* = 8.4, 6.9, 1.5 Hz, 1H, C5), 7.75-7.72 (m, 2H, C6, C13), 7.54 (t, *J* = 7.9 Hz, 1H, C14-H); **^{13}C NMR** (126 MHz; DMSO-d_6): δ 168.0 (C1), 154.6 (Cq^{Ar}), 148.7 (Cq^{Ar}), 140.6 (Cq^{Ar}), 138.5 (Cq^{Ar}), 133.1 (C^{Ar}), 131.6 (C14), 130.9 (C5), 130.33 (C4), 130.17 (C11), 128.6 (C^{Ar}), 126.7 (C15), 125.8 (C7), 124.1 (Cq^{Ar}), 123.0 (Cq^{Ar}), 119.6 (C16); **HRMS** *m/z* (ESI+) $C_{16}H_{11}BrNO_2$ calculated $[M+H]^+$ 327.9973, found 327.9987.

6-chloro-2-(pyridin-3-yl)quinoline-4-carboxylic acid (31)

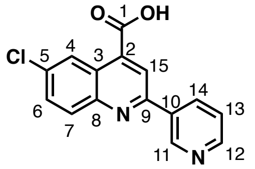

Using the general procedure, 5-chloroisatin (500 mg, 2.75 mmol), 3-acetylpyridine (0.35 mL, 3.45 mmol) and KOH (464 mg, 8.27 mmol) were combined yielding the crude product, which was subsequently recrystallised from acetone to yield the pure product (61 mg, 12%). **IR** (solid) ν_{max} 3100-2800 (O-H_{acid}, broad), 1710 (C=O_{acid}, sharp), 1611 (C-$O_{aromatic}$, sharp), 1592 (C=$C_{aromatic}$, sharp),

cm^{-1}; ^{1}**H NMR** (500 MHz; DMSO-d$_6$): δ 9.46 (s, 1H, C11-H), 8.79 (d, *J* = 2.3 Hz, 1H, C4-H), 8.74 (d, *J* = 4.1 Hz, 1H, C12-H), 8.65 (ddd, *J* = 8.0, 2.3, 1.7 Hz, 1H, C14-H), 8.61 (s, 1H, C15-H), 8.23 (dd, *J* = 9.0, 0.4 Hz, 1H, C7-H), 7.91 (dd, *J* = 9.0, 2.4 Hz, 1H, C6-H), 7.61 (ddd, *J* = 8.0, 4.8, 0.5 Hz, 1H, C13-H); ^{13}**C NMR** (126 MHz; DMSO-d$_6$): δ 167.0 (C1), 154.5 (Cq^{Ar}), 150.9 (C12), 148.4 (Cq^{Ar}), 148.4 (C11), 147.0 (Cq^{Ar}), 134.8 (C14), 133.1 (Cq^{Ar}), 132.9 (Cq^{Ar}), 132.0 (C7), 131.0 (C6), 124.5 (Cq^{Ar}), 124.4 (C4), 124.0 (C13), 120.6 (C15); **HRMS** *m/z* (ESI+) $C_{15}H_{10}ClN_2O_2$ calculated $[M+H]^+$ 285.0431, found 285.0462.

2-(3,5-difluorophenyl)quinoline-4-carboxylic acid (32)

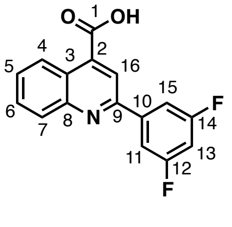

Using the general procedure, isatin (147 mg, 1.0 mmol), 3,5-difluoroacetophenone (188 mg, 1.2 mmol) and KOH (168 mg, 3.0 mmol) were combined yielding the crude product, which was subsequently recrystallised from acetone to yield the pure product (157 mg, 55%).

IR (solid) ν_{max}, 3100-2550 (O-H$_{acid}$, broad), 1686 (C=O$_{acid}$, sharp), 1587 (C=C$_{aromatic}$, sharp), cm^{-1}; ^{1}**H NMR** (500 MHz; DMSO-d$_6$): δ 8.62 (dm, *J* = 8.5, 0.8 Hz, 1H), 8.52 (s, 1H), 8.20 (ddd, *J* = 8.5, 1.3, 0.6 Hz, 1H), 8.07-8.03 (m, 2H), 7.89 (ddd, *J* = 8.4, 6.9, 1.5 Hz, 1H), 7.75 (ddd, *J* = 8.4, 6.9, 1.4 Hz, 1H), 7.43 (tt, *J* = 9.1, 2.3 Hz, 1H); ^{13}**C NMR** (126 MHz; DMSO-d$_6$): δ 167.5 (C1), 163.0 (dd, *J* = 246.0, 13.2 Hz, C12, C14), 153.2 (t, *J* = 3.0 Hz, C9), 148.1 (Cq^{Ar}), 141.6 (t, *J* = 9.2 Hz, C10), 138.3 (Cq^{Ar}), 130.6 (C5), 129.9 (C4), 128.5 (C6), 125.4 (C7), 123.8 (Cq^{Ar}), 119.0 (C16), 110.4(dd, *J* = 26.9, 6.4 Hz, C11, C15), 105.3 (t, *J* = 25.8 Hz, C13); **HRMS** *m/z* (ESI+) $C_{16}H_{10}F_2NO_2$ calculated $[M+H]^+$ 286.0680, found 286.0674.

2-(3-chloro-5-fluorophenyl)quinoline-4-carboxylic acid (33)

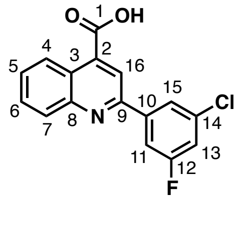

Using the general procedure, isatin (250 mg, 1.70 mmol), 3-chloro-5-fluoroacetophenone (352 mg, 2.03 mmol) and KOH (286 mg, 5.1 mmol) were combined yielding the crude product, which was subsequently recrystallised from acetone to yield the pure product (61 mg, 12%). **IR** (solid) ν_{max} 3150-2500 (O-H$_{acid}$, broad), 1685 (C=O$_{acid}$, sharp), 1584 (C=C$_{aromatic}$, sharp), cm^{-1}; ^{1}**H NMR** (500 MHz; DMSO-d$_6$): δ 14.08 (s, 1H, COO-H), 8.62 (ddd, *J* = 8.5, 1.3, 0.5 Hz, 1H, C7-H), 8.53 (s, 1H, C16-H), 8.26 (t, *J* = 1.5 Hz, 1H, C15-H), 8.21 (ddd, *J* = 8.5, 1.3, 0.6 Hz, 1H, C4-H), 8.16 (ddd, *J* = 10.1, 2.4, 1.5 Hz, 1H, C11-H), 7.89 (ddd, *J* = 8.4, 6.9, 1.5 Hz, 1H, C5-H), 7.75 (ddd, *J* = 8.4, 6.9, 1.4 Hz, 1H, C6-H), 7.62 (dt, *J* = 8.5, 2.1 Hz, 1H, C13-H); ^{13}**C NMR** (126 MHz; DMSO-d$_6$): δ 167.5(C1), 162.6 (d, *J* = 247.3 Hz, C12), 153.1 (Cq^{Ar}), 148.1 (Cq^{Ar}), 141.6 (d, *J* = 8.5 Hz, Cq^{Ar}), 138.4 (Cq^{Ar}), 134.8 (d, *J* = 11.2 Hz, Cq^{Ar}), 130.6 (C5), 129.9 (C4), 128.5 (C6), 125.4 (C7), 123.8 (Cq^{Ar}), 123.3 (d, *J* = 2.8 Hz, C15), 119.1 (C16), 117.3 (d, *J* = 25.1 Hz, C13), 113.1 (d, *J* = 23.4 Hz, C11); **HRMS** *m/z* (ESI+) $C_{16}H_{10}ClFNO_2$ calculated $[M+H]^+$ 302.0384, found 302.0401.

2-(3-(trifluoromethyl)phenyl)quinoline-4-carboxylic acid (34)

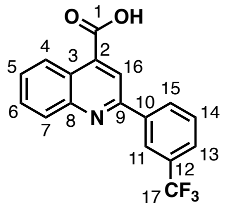

Using the general procedure, isatin (250 mg, 1.70 mmol), 3-(trifluoromethyl)-fluoroacetophenone (0.26 mL, 2.03 mmol) and KOH (286 mg, 5.1 mmol) were combined yielding the crude product, which was subsequently recrystallised from 90% EtOH in H_2O to yield the pure product (85 mg, 16%). **IR** (solid) ν_{max} 3150-2700 (O-H_{acid}, broad), 1718 (C=O_{acid}, sharp), 1583 (C=$C_{aromatic}$, sharp), cm^{-1}; **^{1}H NMR** (500 MHz; DMSO-d_6): δ 14.07 (s, 1H, COO-H), 8.66-8.64 (m, 2H, C7-H, C11-H), 8.61 (dd, *J* = 7.8, 0.6 Hz, 1H, C15-H), 8.57 (s, 1H, C16-H), 8.22 (ddd, *J* = 8.5, 1.3, 0.6 Hz, 1H, C4-H), 7.91-7.87 (m, 2H, C5-H, C13-H), 7.82 (t, *J* = 7.8 Hz, 1H, C14-H), 7.75 (ddd, *J* = 8.4, 6.9, 1.4 Hz, 1H, C6-H); **^{13}C NMR** (126 MHz; DMSO-d_6): δ 167.5 (C1), 154.2 (Cq^{Ar}), 148.3 (Cq^{Ar}), 138.9 (Cq^{Ar}), 138.2 (Cq^{Ar}), 131.3 (C15), 130.5 (C5), 130.2 (C14), 129.9 (C4), 129.8 (q, *J* = 31.9 Hz, C12), 128.27 (C6), 126.4 (q, *J* = 3.6 Hz, C13), 125.4 (C7), 124.2 (q, *J* = 272.4 Hz, C17), 123.7 (Cq^{Ar}), 123.6 (q, *J* = 3.7 Hz, C11), 119.1 (C16); **HRMS** *m/z* (ESI+) $C_{17}H_{11}F_3NO_2$ calculated $[M+H]^+$ 318.0742, found 318.0779.

methoxy-*N*-methyl-3-(trifluoromethyl)benzamide (45)

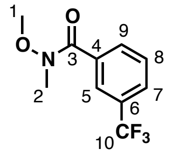

To an oven-dried round-bottomed flask was added Et_3N (3 mL) and N,O-dimethylhydroxyamine hydrochloride (731 mg, 7.5 mmol) in dry DCM (5 mL). Solution of 3-(trifluoromethyl)benzoyl chloride (0.75 mL, 5.0 mmol) in dry DCM (3 mL) was added dropwise into ice-cooled reaction mixture which was allowed to warm up to RT and stirred for 18 h. The reaction was quenched by addition of H20 (10 mL). The organic layer was washed with (sat.) $NaHCO_3$, brine, dried ($MgSO_4$), filtered and the solvent was removed under reduced pressure. The crude product was purified by flash column chromatography (100% DCM) to yield the product (1.046 g, 90%). **R_f** (DCM) = 0.26; **IR** (solid) ν_{max}, 1639 (C=O_{amide}, sharp), 1591 (C=$C_{aromatic}$, sharp), cm^{-1}; **^{1}H NMR** (500 MHz; $CDCl_3$): δ 7.96 (s, 1H, C5-H), 7.88 (d, *J* = 7.8 Hz, 1H, C9-H), 7.70 (dddd, *J* = 7.8, 1.8, 1.1, 0.7 Hz, 1H, C7-H), 7.53 (tt, *J* = 7.8, 0.7 Hz, 1H, C8-H), 3.53 (s, 3H, C2-H_3), 3.37 (s, 3H, C1-H_3); **^{13}C NMR** (126 MHz; $CDCl_3$): δ 168.3 (C3), 134.8 (Cq^{Ar}), 131.6 (C^{Ar}), 130.5 (q, *J* = 32.8 Hz, C6), 128.6 (C^{Ar}), 127.2 (q, *J* = 3.7 Hz, C5), 125.3 (q, *J* = 3.8 Hz, C7), 123.8 (q, *J* = 272.6 Hz, C10), 61.2 (C2), 33.5 (C1); **HRMS** *m/z* (ESI+) $C_{10}H_{11}F_3NO_2$ calculated $[M+H]^+$ 234.0742, found 234.0764.

2-phenyl-1-(3-(trifluoromethyl)phenyl)ethanone (46)

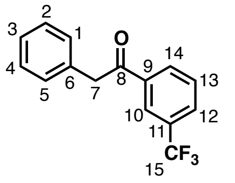

Mg filings (344 mg, 14.15 mmol) in dry Et_2O (10 mL) were added to an oven-dried round-bottomed flask. Benzyl bromide (1.12 mL, 9.43 mmol) was added dropwise into the solution at RT and the reaction mixture was heated under reflux for 1h. After it was allowed to cool to RT, the solution was added dropwise to ice-cold solution of **45** (1100 mg, 4.72 mmol) dissolved in dry Et_2O in an oven-dried round-bottomed flask. The reaction mixture was allowed to warm up to RT and stirred overnight. The mixture was cooled on ice and HCl (3N) was added carefully to quench the reaction. The organic layer was washed with brine, dried ($MgSO_4$), filtered and the solvent was removed under reduced pressure. The crude product was purified by flash column chromatography (20% Hexane in DCM) to yield the desired product (943 mg, 87%). **R_f** (50% DCM / 50% Hexane) = 0.44; **IR** (solid) ν_{max}, 1688 ($C{=}O_{ketone}$, sharp), 1611 ($C{=}C_{aromatic}$, sharp), 1591 ($C{=}C_{aromatic}$, sharp), cm^{-1}; **^{1}H NMR** (500 MHz; $CDCl_3$): δ 8.27 (tt, *J* = 1.6, 0.8 Hz, 1H, C10-H), 8.18 (dm, *J* = 7.8, 0.6 Hz, 1H, C14-H), 7.81 (dm, *J* = 7.1 Hz, 1H, C12-H), 7.60 (tt, *J* = 7.8, 0.6 Hz, 1H, C13-H), 7.37-7.33 (m, 2H, C4-H, C2-H), 7.30-7.27 (m, 3H, C1-H, C3-H, C5-H), 4.32 (s, 2H, C7-H_2). **^{13}C NMR** (126 MHz; $CDCl_3$): δ 196.2 (C8), 137.0 (Cq^{Ar}), 133.8 (Cq^{Ar}), 131.7 (C14), 131.3 (q, *J* = 33.0 Hz, C11), 129.6 (q, *J* = 3.6 Hz, C12), 129.4 (C1, C5), 129.4 (C13), 128.8 (C2, C4), 127.2 (C3), 125.4 (q, *J* = 3.8 Hz, C10), 123.65 (q, *J* = 272.6 Hz, C15), 45.64 (C7); **HRMS** *m/z* (ESI+) $C_{15}H_{12}F_3O$ calculated $[M+H]^+$ 265.0840, found 265.1461.

1-methyl-3-phenylisoquinoline (48)

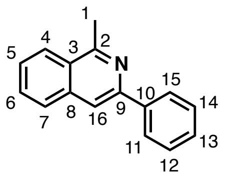

2-phenylacetophenone (2.96 g, 15.1 mmol) was dissolved in dry acetonitrile (125 mL) contained in oven-dried round-bottomed flask. Phosphoryl chloride (9.0 mL, 90.6 mmol) was added dropwise at RT and the solution was refluxed for 72 h. The solvent was removed under reduced pressure, the solid residue was redissolved in H_2O, basified with NaOH (2N) and extracted with DCM. The organic layer was washed with brine, dried ($MgSO_4$), filtered and the solvent was removed under reduced pressure. The crude product was purified by flash column chromatography (3% MeOH in DCM) to yield colourless oil (882 mg, 27%). **R_f** (35 % hexane in DCM) = 0.23; **IR** (solid) ν_{max}, 1621 ($C{=}N_{aromatic}$, sharp), 1579 ($C{=}C_{aromatic}$, sharp), cm^{-1}; **^{1}H NMR** (500 MHz; $CDCl_3$): δ 8.16-8.12 (m, 3H, C7-H, C11-H, C15-H), 7.93 (s, 1H, C16-H), 7.86 (d, *J* = 8.2 Hz, 1H, C4-H), 7.67 (ddd, *J* = 8.2, 6.9, 1.2 Hz, 1H, C5-H), 7.57 (ddd, *J* = 8.3, 6.9, 1.3 Hz, 1H, C6-H), 7.52-7.49 (m, 2H, C12-H, C14-H), 7.42-7.39 (m, 1H, C13-H), 3.05 (d, *J* = 0.5 Hz, 3H, C1-H_3); **^{13}C NMR** (126 MHz; $CDCl_3$): δ 158.7 (Cq^{Ar}), 150.2 (Cq^{Ar}), 140.0 (Cq^{Ar}), 136.9 (Cq^{Ar}), 130.2 (C5), 128.9 (C12, C14), 128.4 (C13), 127.8 (C4), 127.1 (C11, C15), 126.9 (C6), 126.7 (Cq^{Ar}), 125.8 (C7), 115.4 (C16), 22.8 (C1); **HRMS** *m/z* (ESI+) $C_{16}H_{14}N$ calculated $[M+H]^+$ 220.1126, found 220.1148. The data was consistent with literature[6].

1-methyl-3-(3-(trifluoromethyl)phenyl)isoquinoline (49)

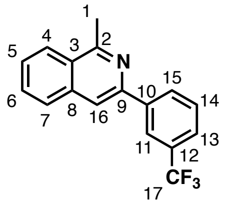

46 (900 mg, 3.41 mmol) was dissolved in dry acetonitrile (25 mL) contained in oven-dried round-bottomed flask. Phosphoryl chloride (1.27 mL, 13.63 mmol) was added dropwise at RT and the solution was refluxed for 72 h. The solvent was removed under reduced pressure, the solid residue was redissolved in H_2O, basified with NaOH (2N) and extracted with DCM. The organic layer was washed with brine, dried (Na_2SO_4), filtered and the solvent was removed under reduced pressure. The crude product was purified by flash column chromatography (50% DCM / 50% Hexane) to yield colourless oil (268 mg, 27%). $\mathbf{R_f}$ (50% DCM / 50% Hexane) = 0.40; **IR** (solid) ν_{max}, 1624 (C=$C_{aromatic}$, sharp), 1594 (C=$C_{aromatic}$, sharp), cm^{-1}; **^{1}H NMR** (500 MHz; $CDCl_3$): δ 8.43 (dd, *J* = 1.5, 0.9 Hz, 1H, C11-H), 8.33 (dm, *J* = 7.6 Hz, 1H, C15-H), 8.15 (dq, *J* = 8.4, 0.9 Hz, 1H, C7), 7.97 (s, 1H, C16-H), 7.89 (dm, *J* = 8.2 Hz, 1H, C4-H), 7.71 (ddd, *J* = 8.1, 6.9, 1.2 Hz, 1H, C5-H), 7.66-7.59 (m, 3H, C6-H, C13-H, C14-H), 3.06 (s, 3H, C1-H_3). **^{13}C NMR** (101 MHz; $CDCl_3$): δ 159.1 (Cq^{Ar}), 148.4 (Cq^{Ar}), 140.7 (Cq^{Ar}), 136.8 (Cq^{Ar}), 131.3 (q, *J* = 32.2 Hz, C12), 130.5 (C5), 130.2 (C15), 129.3 (C^{Ar}), 127.88 (s, I), 127.5 (C4), 127.1 (C^{Ar}), 125.9 (Cq^{Ar}), 125.0 (q, *J* = 3.7 Hz, C13), 124.5 (q, *J* = 272.4 Hz, C17), 124.0 (q, *J* = 3.9 Hz, C11), 115.8 (C16), 22.8 (C1); **HRMS** *m/z* (ESI+) $C_{17}H_{13}F_3N$ calculated $[M+H]^+$ 288.1000, found 288.1019.

3-phenylisoquinoline-1-carboxylic acid (50)

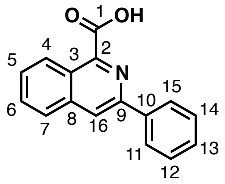

48 (380 mg, 1.6 mmol), N-Bromosuccinimide (850 mg, 4.8 mmol) and benzoyl peroxide (30 mg, 0.12 mmol) were dissolved in CCl_4 (10 mL) and heated under reflux for 20 h. The insoluble material was filtered off through Celite, the filtrate was washed with $NaHCO_3$ (5 %) and the solvent was removed under reduced pressure to yield orange oil that was utilized without further purification. The oil residue was dissolved in a mixture of THF (4 mL) and EtOH (8 mL) and heated to reflux. Solution of $AgNO_3$ (1.09 g, 6.40 mmol) in H_2O (2 mL) was added, the reaction mixture was heated under reflux for 1 h and the insoluble material was removed on Celite while still hot. The filtrate was evaporated to dryness and utilised without further purification. The solid was dissolved in EtOH (5 mL) and treated with solution of $AgNO_3$ (678 mg, 4 mmol) in H_2O (1.5 mL). A solution of NaOH (600 mg, 15 mmol) in H_2O (8 mL) was added dropwise and the reaction mixture was stirred overnight. The insoluble material was removed by filtration through Celite pad, washed with Et_2O on filter and the filtrate was acidified with (conc.) HCl. The filtrate was reduced to 1/3 volume under reduced pressure and ice-cooled. The yellow precipitate was collected, washed with H_2O on filter and triturated with Et_2O to yield the product as yellow solid (195 mg, 49%). **IR** (solid) ν_{max}, 3200-2850 (O-H_{acid}, broad), 1752 (C=O_{acid}, sharp), 1621 (C=$N_{aromatic}$, sharp), 1583 (C=$C_{aromatic}$, sharp), cm^{-1}; **^{1}H NMR** (500 MHz; DMSO-d_6): δ 13.72 (s, 1H, COO-H), 8.64 (s, 1H, C16-H), 8.51 (dq, *J* = 8.5, 1.0 Hz, 1H, C7-H),

8.28-8.25 (m, 2H, C11-H, C15-H), 8.14 (dm, *J* = 8.3 Hz, 1H, C4-H), 7.86 (ddd, *J* = 8.2, 6.9, 1.2 Hz, 1H, C5-H), 7.75 (ddd, *J* = 8.4, 7.0, 1.4 Hz, 1H, C6-H), 7.55 (tm, *J* = 7.6 Hz, 2H, C12-H, C14-H), 7.48-7.45 (m, 1H, C13-H). **^{13}C NMR** (126 MHz; DMSO-d$_6$): δ 167.4 (C1), 150.4 (Cq^{Ar}), 148.4 (Cq^{Ar}), 137.9 (Cq^{Ar}), 137.4 (Cq^{Ar}), 131.1 (C5), 129.0 (C13), 128.9 (C12, C14), 128.6 (C6), 127.8 (C4), 126.7 (C11, C15), 125.8 (C7), 124.2 (Cq^{Ar}), 118.9 (C16); **HRMS** *m/z* (ESI+) $C_{16}H_{12}NO_2$ calculated [M+H]$^+$ 250.0868, found 259.0881. The data was consistent with literature[6].

3-(3-(trifluoromethyl)phenyl)isoquinoline-1-carboxylic acid (51)

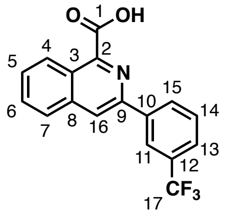

49 (65 mg, 0.23 mmol), *N*-Bromosuccinimide (120 mg, 0.68 mmol) and benzoyl peroxide (4 mg, 0.02 mmol) were dissolved in CCl_4 (5 mL) and heated under reflux for 20 h. The insoluble material was filtered off through Celite, the filtrate was washed with $NaHCO_3$ (5%) and the solvent was removed under reduced pressure to yield oil that was utilized without further purification. The oil residue was dissolved in a mixture of THF (2 mL) and EtOH (6 mL) and heated to reflux. Solution of $AgNO_3$ (156 mg, 0.92 mmol) in H_2O (1 mL) was added, the reaction mixture was heated under reflux for 1 h and the insoluble material was removed on Celite while still hot. The filtrate was evaporated to dryness and utilized without further purification. The solid was dissolved in EtOH (3 mL) and treated with solution of $AgNO_3$ (98 mg, 0.58 mmol) in H_2O (1 mL). A solution of NaOH (88 mg, 2.2 mmol) in H_2O (2 mL) was added dropwise and the reaction mixture was stirred overnight. The insoluble material was removed by filtration through Celite pad, washed with Et_2O on filter and the filtrate was acidified with (conc.) HCl. The filtrate was reduced to 1/3 volume under reduced pressure and ice-cooled. The yellow precipitate was collected, washed with H_2O on filter and triturated with Et_2O to yield the product as yellow solid (21 mg, 28%). **IR** (solid) v_{max}, 1755 (C=O$_{ketone}$, sharp), 1624 (C=C$_{aromatic}$, sharp), 1591 (C=C$_{aromatic}$, sharp), cm^{-1}; **^{1}H NMR** (500 MHz; DMSO-d$_6$): δ 13.80 (s, 1H, COO-H), 8.83 (s, 1H, C16-H), 8.62 (s, 1H C11-H), 8.59 (d, *J* = 7.6 Hz, 1H, C15-H), 8.56 (dd, *J* = 8.5, 0.8 Hz, 1H, C7-H), 8.17 (d, *J* = 8.2 Hz, 1H, C4-H), 7.90 (ddd, *J* = 8.2, 6.9, 1.2 Hz, 1H, C5-H), 7.85-7.78 (m, 3H, C13-H, C14-H, C6-H); **^{13}C NMR** (126 MHz; DMSO-d$_6$): δ 167.2 (s, Q), 150.6 (C10), 146.5 (Cq^{Ar}), 138.9 (Cq^{Ar}), 137.4 (Cq^{Ar}), 131.3 (C5), 130.5 (C15), 130.1 (C14), 129.8 (q, *J* = 31.5 Hz, C12), 129.15 (C6), 128.0 (C4), 125.9 (C7), 125.5 (q, *J* = 3.5 Hz, C13), 124.5 (Cq^{Ar}), 124.3 (q, *J* = 272.7 Hz, C17), 123.1 (q, *J* = 3.5 Hz, C11), 119.8 (C16); **HRMS** *m/z* (ESI+) $C_{17}H_{11}F_3NO_2$ calculated [M+H]$^+$ 318.0742, found 318.0756.

Supplementary Information References

1. Wang, Z.-X. An exact mathematical expression for describing competitive binding of two different ligands to a protein molecule. *FEBS Lett.* **360,** 111–114 (1995).
2. Nikolovska-Coleska, Z. *et al.* Development and optimization of a binding assay for the XIAP BIR3 domain using fluorescence polarization. *Anal. Biochem.* **332,** 261–273 (2004).
3. Elling, R. A. *et al.* Mouse Aurora A: expression in Escherichia coli and purification. *Protein Expr. Purif.* **54,** 139–146 (2007).
4. Peränen, J., Rikkonen, M., Hyvönen, M. & Kääriäinen, L. T7 vectors with a modified T7lac promoter for expression of proteins in Escherichia coli. *Anal. Biochem.* **236,** 371–373 (1996).
5. Pettersen, E. F. *et al.* UCSF Chimera--a visualization system for exploratory research and analysis. *J. Comput. Chem.* **25,** 1605–1612 (2004).
6. Giardina, G. A. *et al.* Discovery of a novel class of selective non-peptide antagonists for the human neurokinin-3 receptor. 1. Identification of the 4-quinolinecarboxamide framework. *J. Med. Chem.* **40,** 1794–1807 (1997).
